# Unraveling immune-inflammation-aging network interactions: an interpretable machine learning model predicts the risk of postherpetic neuralgia

**DOI:** 10.3389/fimmu.2026.1802320

**Published:** 2026-06-12

**Authors:** Pei-pei Kang, Shi-jie Bi, Yan-ran Kang, She-jiao Han, Guo-xian Kang, Fei Zhao

**Affiliations:** 1 Pain Department, The Second Affiliated Hospital of Henan University of Science and Technology, Luoyang, Henan, China; 2 First Clinical Medical College, Shandong University of Traditional Chinese Medicine, Jinan, Shandong, China; 3 Nursing Major, Medical College, Sias University, Zhengzhou, Henan, China; 4 Pain Department, Yichuan County People’s Hospital, Luoyang, China; 5 Applied Psychology, Gansu Minzu Normal University, Hezuo, Gansu, China; 6 Gerontology Department, The First Affiliated Hospital of Shandong First Medical University & Shandong Provincial Qianfoshan Hospital, Jinan, Shandong, China

**Keywords:** immune-inflammation-aging network, postherpetic neuralgia, predictive model, SHAP, XGBoost

## Abstract

**Background:**

Postherpetic neuralgia (PHN) is the most common and intractable complication of herpes zoster (HZ). Early and accurate identification of patients at high risk for PHN is crucial for effective interventions. This study aimed to establish a high-performance and interpretable machine learning prediction model.

**Methods:**

This was a single-center retrospective cohort study that ultimately included 480 patients hospitalized with a diagnosis of HZ at the First Affiliated Hospital of Shandong First Medical University (January to December 2024). Patients were divided into PHN and non-PHN groups. Multidimensional predictors were collected through the electronic medical record system. Integrated feature screening was performed using the Boruta algorithm, random forest, and LASSO regression. Six machine learning models (including XGBoost) were trained and compared using nested cross-validation, and the optimal model was interpreted via the SHAP framework.

**Results:**

The incidence of PHN was 23.3%. Eight key predictors were identified: age, neutrophil-to-lymphocyte ratio (NLR), absolute lymphocyte count (ALC), serum albumin (ALB), platelet-to-lymphocyte ratio (PLR), absolute eosinophil count (AEC), serum calcium (Ca) and neutrophil-to-platelet ratio (NPR). Among the six models, XGBoost demonstrated optimal performance with an AUC of 0.919 (95% CI: 0.910–0.927) in nested cross-validation. It also showed a sensitivity of 0.836 and a specificity of 0.831. SHAP analysis suggested relatively linear effects for age, ALB, ALC and AEC, whereas NLR, PLR and Ca exhibited more complex, potentially nonlinear associations with PHN risk. Interaction analysis further indicated extensive synergistic effects among these factors, collectively providing a preliminary outline of a potential risk network grounded in “aging,” centered on “immune-inflammation,” and modulated by nutritional status. An online calculator based on this model has been deployed.

**Conclusions:**

This study developed and internally validated a high-performance, interpretable PHN risk prediction model. Its interpretable output suggests that the occurrence of PHN may be associated with an imbalance in the immune-inflammation-aging network, generating new hypotheses for its pathogenesis. The accompanying online tool offers research-grade decision support for individualized clinical risk management, pending external validation.

## 1 Introduction

Postherpetic neuralgia (PHN) is a chronic neuropathic pain that persists for more than one month after herpes zoster (HZ) healing, and it is the most common and serious complication of HZ (1). It is mainly manifested as paroxysmal burning, electric shock-like, needle-like, or knife-like pain in the skin lesion area, accompanied by local skin allodynia and hyperalgesia, which can lead to anxiety, depression, insomnia, and other psychiatric symptoms. Some patients may even experience suicidal thoughts and behaviors (2). PHN not only seriously affects patients’ quality of life but also increases the medical burden (3, 4). A systematic review integrating the incidence of HZ and PHN in different regions of the world showed that the incidence of PHN in HZ patients ranged from 5% to 30%, and more than 30% of PHN patients had pain for more than one year (5). In China, a nationwide cross-sectional study reported an HZ prevalence of 5.85‰, and the incidence of PHN among HZ patients was 15.84%, with a higher proportion among women and the elderly (6). Existing PHN treatment options include drug therapy (calcium channel modulators, tricyclic antidepressants, opioid analgesics, etc.), interventional therapy (nerve block, pulsed radiofrequency, radiofrequency thermocoagulation, spinal cord stimulation, etc.) (2, 7–9), and traditional Chinese medicine treatment (acupuncture, Chinese herbal medicine, pricking and cupping therapy, etc.) (10–12). However, these treatments face challenges such as variable efficacy, drug side effects, and accessibility. Therefore, early identification of individuals at high risk for PHN in the acute phase of HZ and implementation of targeted interventions to prevent its occurrence are of critical clinical importance.

Existing studies have identified several potential risk factors for PHN. In addition to advanced age—the most well-defined risk factor—rash severity, prodromal pain, unhealthy lifestyle, immunosuppressive status, and comorbidities such as diabetes, chronic obstructive pulmonary disease, hypertension, malignant tumours, or chronic kidney disease have also been associated with PHN development (13–16). In recent years, the role of systemic inflammation in the development of PHN has attracted increasing attention. Some peripheral blood inflammatory markers, such as C-reactive protein (CRP), neutrophil-to-lymphocyte ratio (NLR), and platelet-to-lymphocyte ratio (PLR), have been initially confirmed to have predictive potential (13, 17–22). However, with the exception of age, the predictive value of most risk factors remains controversial. Furthermore, most existing studies are based on linear models, which fail to capture the complex nonlinear interactions among biomarkers. This limitation not only hinders the elucidation of the potential multi-system interactive risk network underlying PHN but also compromises the accuracy and robustness of prediction models.

With the development of artificial intelligence, machine learning has shown potential in building early predictive models for PHN. However, methodological aspects of research in this field require further refinement. Specifically, at the technical level, feature screening strategies, validation of complex nonlinear models, and rigorous frameworks to ensure model generalizability need to be improved. At the variable inclusion level, selection criteria are not standardized, leading to significant heterogeneity. At the application level, transforming high-performance models into convenient clinical decision-support tools remains a key challenge.

Based on this, the present study aims to systematically collect multidimensional clinical indicators through a retrospective cohort study. First, the random forest, LASSO, and Boruta algorithms were integrated to perform robust feature selection. Subsequently, a nested cross-validation framework was employed for model training and hyperparameter tuning, and various machine learning models were compared on a test set to develop a high-performance predictive model, followed by internal validation. Concurrently, the SHAP interpretability framework is applied to gain an in-depth understanding of the optimal model, unveiling the contribution patterns and interaction effects of key variables, along with a sensitivity analysis to assess model robustness. Finally, the model was deployed as an online calculator to provide a quantitative tool and decision-making support for the early clinical identification of high-risk patients and their individualized prevention and management. This exploratory study aims not only to develop a high-accuracy prediction model but also to generate, through explainable artificial intelligence methods, testable hypotheses regarding the key biomarker interaction network that may underlie PHN occurrence. This approach is expected to provide preliminary insights into the potential pathological mechanism of a putative “immune-inflammation-aging” multidimensional imbalance.

## 2 Methods

**Figure 1:**
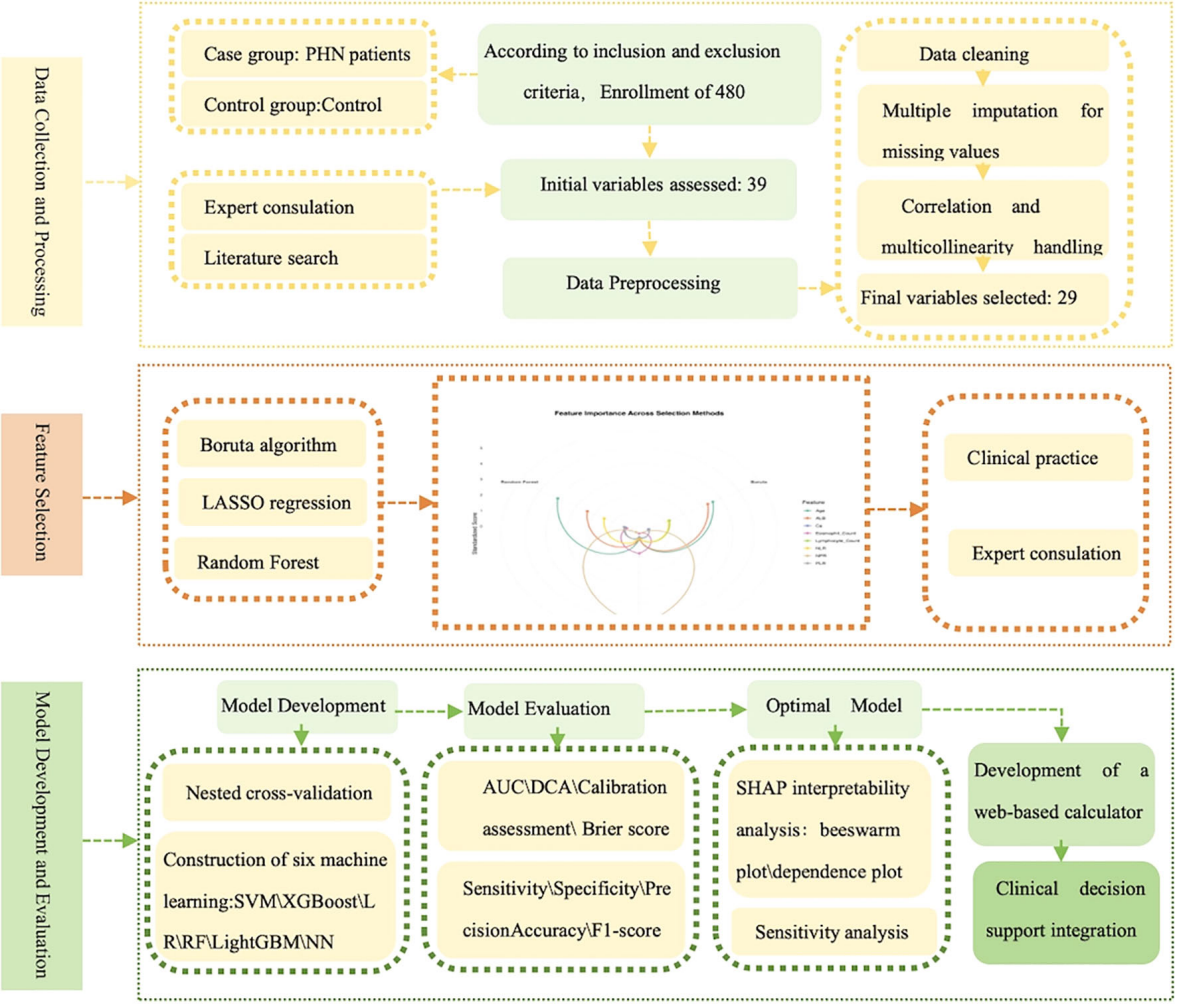
Flowchart.

This was a retrospective cohort study aimed at developing and validating a predictive model for PHN. The study was conducted in strict accordance with the Declaration of Helsinki and was approved by the Ethics Committee of the First Affiliated Hospital of Shandong First Medical University & Shandong Provincial Qianfoshan Hospital (Approval No: YXLL-KY-20259(045)). **Figure 1** presents the study flowchart.

### 2.1 Study design and participants

This was a single-center, retrospective cohort study conducted at the First Affiliated Hospital of Shandong First Medical University & Shandong Provincial Qianfoshan Hospital. Adult patients (age ≥ 18 years) who were hospitalized with a diagnosis of HZ between January 1, 2024, and December 31, 2024 were consecutively enrolled. The diagnosis of PHN was comprehensively determined by combining telephone follow-ups, outpatient review records, and rehospitalization records.

Inclusion criteria: (1) Meeting the clinical diagnostic criteria for HZ; (2) age ≥ 18 years; (3) Having received no prior systemic antiviral or interventional pain therapy for the current herpes episode before hospitalization.

Exclusion criteria: (1) Comorbidities that could interfere with sensory or pain assessment, such as psychiatric disorders or severe neurological diseases; (2) Coexisting other chronic pain conditions that might confound the determination of PHN; (3) Lack of complete key blood biochemical test results in the inpatient medical records.

Sample size calculation: This study planned to include approximately eight candidate predictor variables. With reference to previous literature, the model discrimination (C-statistic) was assumed to be 0.8 (23, 24), and the incidence rate was 15.84% (6). Estimation was performed using the pmsampsize package in R software (version 4.2.3). The parameters were set as follows: type = “b” (for binary outcome), parameters = 8 (number of predictors), prevalence = 0.1584 (incidence rate), cstatistic = 0.8 (C-statistic), and dropout_rate = 0.10 (dropout rate). The calculation yielded a minimum required sample size of 420 cases. Considering a 10% dropout rate, the minimum total sample size was set at 467 cases.

### 2.2 Definition of study outcome

The primary outcome was PHN, defined as pain persisting for ≥ 1 month after rash healing in the original dermatomal area, consistent with the Chinese Expert Consensus on the Diagnosis and Treatment of PHN (1). The outcome was assessed independently by two chief physicians from the Department of Dermatology and the Department of Pain Medicine. A comprehensive assessment was performed based on structured follow-up questionnaires, outpatient records, and rehospitalization medical records. Any discrepancies between the two assessors were resolved through discussion to reach a consensus.

Due to the retrospective nature of this study, the following methodological limitations should be noted: (1) standardized and validated neuropathic pain scales (e.g., ID Pain, DN4) were not used; (2) quantitative pain intensity measurements (e.g., NRS or VAS) were not performed; and (3) the follow-up time point was approximately one month after rash healing, without a pre-specified uniform time window. These limitations may introduce recall bias and outcome misclassification, which are inherent to retrospective designs.

### 2.3 Predictor variables and data collection

Based on a review of previous literature and discussions with multidisciplinary experts (covering dermatology, pain medicine, and geriatrics), 39 predictor variables were initially identified, spanning the following dimensions:

Demographic and baseline characteristics: age, gender, and body mass index (BMI).

Clinical characteristics: time from onset to hospital visit, lifestyle factors (history of smoking and alcohol consumption), and recent medication history (use of glucocorticoids and/or immunosuppressants within ≤ 3 months).

Disease characteristics: presence of prodromal pain, affected nerve segments, and occurrence of herpes infection.

Comorbidities: history of cancer, history of diabetes, history of autoimmune disease, and Charlson Comorbidity Index (CCI) score.

Treatment measures: use of corticosteroids, antiviral drugs, calcium channel modulators, neurotrophic drugs, non-steroidal anti-inflammatory drugs (NSAIDs), and opioids.

Serological indices and derived ratios: HbA1c, serum albumin (ALB), serum calcium (Ca), triglyceride (TG), white blood cell count (WBC), absolute neutrophil count (ANC), absolute lymphocyte count (ALC), absolute monocyte count (AMC), absolute eosinophil count (AEC), absolute basophil count (ABC), hemoglobin (Hb), platelet count (PLT), CRP, lymphocyte-to-monocyte ratio (LMR), NLR, PLR, neutrophil-to-platelet ratio (NPR), and platelet-to-albumin ratio (PAR).

All data were independently extracted from the hospital’s electronic medical record system by two researchers and cross-checked for accuracy. Detailed definitions for some included variables are provided in **Supplementary Materials Table 1**.

### 2.4 Statistical analysis

All statistical analyses and machine learning modeling in this study were performed using R software (version 4.3.3; https://www.R-project.org). Multiple R packages were utilized, including mice, car, OutliersO3, caret, VIM, glmnet, randomForest, mlr3, xgboost, e1071, stats, nnet, lightgbm, pROC, rms, rmda, MLmetrics, and shapviz.

Data preprocessing: Continuous variables were standardized. Categorical variables (including binary and multi-class variables) were encoded numerically.

Data presentation: Continuous variables are presented as mean ± standard deviation (SD) if normally distributed or as median (interquartile range) if non-normally distributed. Categorical variables are expressed as frequency (percentage).

Between-group comparisons: To preliminarily describe the association between predictor variables and the outcome, the following tests were employed: Student’s *t*-test was used for continuous variables that were normally distributed with homogeneity of variance; otherwise, the Mann-Whitney U test was applied. For categorical variables, the chi-square test or Fisher’s exact test (when any expected cell count was < 5) was used. All tests were two-tailed, and a *p*-value < 0.05 was considered statistically significant.

### 2.5 Data processing and cleaning

A systematic cleaning and preprocessing pipeline was applied to the initially collected 39 predictor variables to ensure data quality and modeling robustness. The procedures were as follows. First, constant variables with zero or near-zero variance were removed. Second, given the known sex differences in hemoglobin levels, hemoglobin was sex-standardized to eliminate the confounding effect of sex. Third, variables with a missing rate exceeding 20% were excluded. Subsequently, missing data in the remaining variables were handled using multiple imputation by chained equations (MICE) to generate 10 complete datasets (detailed imputation specifications, including convergence diagnostics and a complete-case sensitivity analysis, are provided in **Supplementary Methods**, **Supplementary Table 2**). Concurrently, potential extreme outliers were identified and then either corrected or removed based on their clinical plausibility. Next, highly correlated variables (defined by an absolute Pearson’s correlation coefficient > 0.8) were identified, and from each pair, the variable with lesser clinical relevance or greater information overlap was removed. Finally, Variance Inflation Factor (VIF) diagnostics were performed on the remaining variables to exclude those with severe multicollinearity (VIF > 10). Following these steps, a final set of 29 variables was retained for subsequent feature selection and model development. (details on excluded variables are provided in **Supplementary Table 3**, and the distribution of missing values and correlations among highly related variables are shown in **Supplementary Figure 1**).

### 2.6 Feature selection and model development

#### 2.6.1 Feature selection

To develop a model with both high predictive performance and clinical interpretability, an integrated feature selection strategy was employed to comprehensively evaluate the importance of each of the 29 candidate variables. First, the imputed dataset was randomly split into a training set and an independent test set at a 7:3 ratio, with all feature selection procedures conducted within the training set to prevent data leakage. Three complementary methods were then applied in parallel: the Boruta algorithm (based on shadow feature comparison), random forest (using mean decrease in accuracy as the importance metric), and LASSO regression. The importance scores from these three methods were normalized and combined into a composite score using equal weights (1:1:1). The top-ranked features were evaluated via 5-fold cross-validation using a logistic regression model.Detailed parameters of the feature selection process, along with feature stability and weight sensitivity analyses, are provided in **Supplementary Table 4**, **Supplementary Methods**.

#### 2.6.2 Model development and performance evaluation

The eight variables selected were used as input features to construct six machine learning prediction models: XGBoost, random forest (RF), support vector machine (SVM), logistic regression (LR), neural network (NN), and LightGBM. To obtain unbiased and robust performance estimates, a rigorous 5 × 5 two-layer nested cross-validation framework (25) was employed on the complete imputed dataset (n = 480). In the outer loop (5-fold), final generalization performance was evaluated: the complete dataset was split into five mutually exclusive folds, and in each iteration, one fold was held out as the test set while the remaining four folds were combined as the training set. The inner loop (5-fold) was conducted within the training set of the outer loop for hyperparameter tuning and selection, also using 5-fold cross-validation. Bayesian optimization was used to efficiently search for the optimal hyperparameter combination within a predefined parameter space. This nested design ensured complete separation between the data used for tuning (inner validation set) and the data used for final evaluation (outer test set), effectively preventing information leakage and optimistic bias. The entire nested cross-validation procedure was independently repeated five times to enhance the stability of the evaluation. Final generalization performance metrics were calculated as the mean values across all outer test sets, with variability expressed as the standard deviation. The reported performance metrics included the area under the receiver operating characteristic curve (AUC), accuracy, precision, sensitivity, specificity, F1 score, and Brier score. Detailed parameters for model construction and the hyperparameter search space for each model are provided in **Supplementary Table 5**.

### 2.7 Model performance comparison and optimal model selection

The performance of all six models was evaluated on the outer test folds of the nested cross-validation. Based on the aggregated prediction results from all outer test sets for each model, receiver operating characteristic (ROC) curves were plotted to calculate the area under the curve (AUC). Calibration curves (calibrated using the Platt scaling method) and the decision curve analysis (DCA) were also performed. The optimal model (XGBoost) was selected for subsequent in-depth analysis based on a comprehensive comparison of key metrics, including AUC and clinical net benefit.

### 2.8 Analysis of interpretability and robustness of the optimal model

#### 2.8.1 Model explanatory analysis based on SHAP framework

To comprehensively evaluate the reliability of the optimal model and the transparency of its decision logic, the SHAP (Shapley Additive exPlanations) framework was used in the study. First, for global interpretation: The overall contribution of each feature was calculated, and a SHAP summary (beeswarm) plot was drawn to visually show the feature importance and the direction of its impact. Second, for dependency effect analysis: SHAP dependence plots were generated to visualize potentially nonlinear relationships between each feature and PHN predictive risk. Third, for interaction exploration: Pairwise interaction effects between the main features were explored using SHAP interaction plots to gain a deeper understanding of the pathogenesis of PHN.

#### 2.8.2 Model robustness and sensitivity analysis

To examine the robustness of the model, a three-dimensional sensitivity analysis was conducted. First, feature importance stability analysis: The model was repeatedly trained on different training subsets (each fold of the cross-validation), and feature importance was recalculated. This process assessed the volatility in the ranking of core predictor importance, thereby confirming the reliability of the features on which the model relies. Second, hyperparameter sensitivity analysis: Within a reasonable grid range, key hyperparameters of the optimal model (such as learning rate and maximum tree depth) were systematically perturbed. Their impact on model performance (with AUC as the primary metric) was observed to evaluate the model’s sensitivity to parameter settings and the stability of its performance. Third, Bootstrap internal validation: The Bootstrap resampling technique (1,000 replicates) was used for internal validation of model performance. The stability and precision of the model’s performance estimates were evaluated by calculating the distribution and variability of performance metrics (such as AUC, Brier score, and predicted probability) across the Bootstrap samples.

### 2.9 Development of a clinical decision support tool

Based on the validated optimal prediction model, an interactive web calculator was developed to facilitate clinical translation. This tool enables clinicians to input patient-specific indicators and obtain an individualized PHN risk prediction promptly, thereby supporting clinical decision-making.

## 3 Results

### 3.1 Baseline characteristics

This study ultimately included 480 patients with HZ. Because the model construction primarily employed nested cross-validation, the baseline characteristics analysis focused mainly on comparing the PHN group with the non-PHN control group (**Table 1**). Among these patients, 112 (23.3%) developed PHN during follow-up, while 368 (76.7%) did not. The incidence of PHN in this study (23.3%) was higher than the 15.4% reported in a national study (6) and also higher than the 19.4% reported for outpatients (26). However, it was similar to the finding of another study involving hospitalized patients (25.4%) (27). These findings suggest that hospitalized patients may have a higher risk of PHN due to more severe clinical symptoms or a greater number of comorbidities. Overall, patients in the PHN group were significantly older than those in the control group (*p* < 0.001) and had a higher proportion of females (*p* = 0.021). Regarding clinical manifestations, the proportion of patients with prodromal pain was significantly higher in the PHN group (*p* = 0.035). No statistically significant difference was observed in the overall distribution of affected spinal segments between the two groups (*p* = 0.189). However, there was a trend towards a higher proportion of C5 to T2 segment involvement in the PHN group than in the control group (15.2% vs. 7.9%). No statistically significant differences were found between the two groups in terms of BMI, time from onset to first hospital visit, distribution of affected segments, history of smoking or alcohol consumption, history of cancer or diabetes, CCI score, or the treatment regimen involving corticosteroids and antiviral drugs (all *p* > 0.05).

**Table 1:** Baseline characteristics of study participants.

Variable	Overall (N = 480)^1^	Control (n = 368)^1^	PHN (n = 112)^1^	*P* value^2^
Age, years				<0.001
Median (Q1, Q3)	61.000 [50.000, 70.000]	60.000 [46.000, 69.000]	67.000 [57.500, 73.000]	
Gender n (%)				0.021
Female	265 (55.2%)	192 (52.2%)	73 (65.2%)	
Male	215 (44.8%)	176 (47.8%)	39 (34.8%)	
BMI, kg/m²				0.696
Median (Q1, Q3)	24.705 [22.660, 27.152]	24.820 [22.593, 27.113]	24.590 [22.878, 27.340]	
First appointment, days n (%)				0.386
<=3	70 (14.6%)	57 (15.5%)	13 (11.6%)	
>3	410 (85.4%)	311 (84.5%)	99 (88.4%)	
Prodromal pain n (%)				0.035
No	115 (24.0%)	97 (26.4%)	18 (16.1%)	
Yes	365 (76.0%)	271 (73.6%)	94 (83.9%)	
Involved spinal segment n (%)				0.189
C1-C4	41 (8.5%)	32 (8.7%)	9 (8.0%)	
C5-T2	46 (9.6%)	29 (7.9%)	17 (15.2%)	
LSR	86 (17.9%)	64 (17.4%)	22 (19.6%)	
T3-T12	223 (46.5%)	176 (47.8%)	47 (42.0%)	
TN	84 (17.5%)	67 (18.2%)	17 (15.2%)	
Smoking history n (%)				0.506
Current smoker	37 (7.7%)	31 (8.4%)	6 (5.4%)	
Former smoker	25 (5.2%)	20 (5.4%)	5 (4.5%)	
Never smoker	418 (87.1%)	317 (86.1%)	101 (90.2%)	
Alcohol history n (%)				0.327
Current drinker	30 (6.2%)	25 (6.8%)	5 (4.5%)	
Former drinker	10 (2.1%)	6 (1.6%)	4 (3.6%)	
Never drinker	440 (91.7%)	337 (91.6%)	103 (92.0%)	
Cancer history n (%)				0.388
No	443 (92.3%)	337 (91.6%)	106 (94.6%)	
Yes	37 (7.7%)	31 (8.4%)	6 (5.4%)	
Diabetes history n (%)				0.091
No	400 (83.3%)	313 (85.1%)	87 (77.7%)	
Yes	80 (16.7%)	55 (14.9%)	25 (22.3%)	
CCI-Score n (%)				0.143
0	404 (84.2%)	316 (85.9%)	88 (78.6%)	
1	66 (13.8%)	46 (12.5%)	20 (17.9%)	
2	10 (2.1%)	6 (1.6%)	4 (3.6%)	
Corticosteroids use n(%)				0.953
No	50 (10.4%)	39 (10.6%)	11 (9.8%)	
Yes	430 (89.6%)	329 (89.4%)	101 (90.2%)	
Antivirals use n (%)				0.418
Combination therapy	455 (94.8%)	351 (95.4%)	104 (92.9%)	
Monotherapy	25 (5.2%)	17 (4.6%)	8 (7.1%)	
ALB, g/L				<0.001
Median (Q1, Q3)	42.800 [40.300, 45.000]	43.200 [40.500, 45.300]	41.500 [39.075, 44.025]	
Ca, mmol/L				0.062
Median (Q1, Q3)	2.300 [2.230, 2.360]	2.300 [2.240, 2.360]	2.280 [2.218, 2.360]	
ANC, ×10^9^/L				0.815
Median (Q1, Q3)	4.410 [3.170, 5.812]	4.450 [3.180, 5.893]	4.320 [3.071, 5.700]	
ALC, ×10^9^/L				0.041
Median (Q1, Q3)	1.610 [1.190, 2.092]	1.630 [1.228, 2.125]	1.445 [1.078, 2.015]	
ABC, ×10^9^/L				0.414
Median (Q1,Q3)	0.020 [0.010, 0.033]	0.020 [0.010, 0.030]	0.020 [0.010, 0.040]	
AEC, ×10^9^/L				0.190
Median (Q1, Q3)	0.025 [0.000, 0.070]	0.030 [0.000, 0.080]	0.020 [0.000, 0.060]	
AMC,×10^9^/L				0.459
Median (Q1, Q3)	0.500 [0.370, 0.650]	0.500 [0.370, 0.633]	0.500 [0.350, 0.682]	
Hb-zsore, g/L				0.206
Mean SD (Q1, Q3)	0.04 (1.23)	0.06(1.29)	-0.06(1.22)	
LMR				0.048
Median (Q1, Q3)	3.418 [2.482, 4.731]	3.604 [2.551, 4.822]	3.105 [2.374, 4.452]	
NLR				0.544
Median (Q1, Q3)	2.708 [1.939, 3.818]	2.708 [1.877, 3.752]	2.707 [2.075, 4.078]	
PLR				0.741
Median (Q1, Q3)	130.086 [97.130, 174.813]	129.136 [97.130, 172.669]	131.815 [96.998, 184.298]	
NPR				0.357
Median (Q1, Q3)	0.021 [0.015, 0.028]	0.021 [0.015, 0.028]	0.023 [0.016, 0.028]	
PAR				0.988
Median (Q1, Q3)	4.955 [4.121, 5.858]	4.897 [4.130, 5.850]	5.050 [4.089, 5.941]	
CRP, mg/L				<0.001
Median (Q1, Q3)	3.110 [3.110, 3.200]	3.110 [3.110, 3.110]	3.110 [3.110, 4.705]	
HbA1c, %				<0.001
Median (Q1, Q3)	5.800 [5.500, 6.300]	5.800 [5.400, 6.125]	6.000 [5.600, 6.525]	
TG, mmol/L				0.083
Median (Q1, Q3)	0.910 [0.700, 1.393]	0.885 [0.680, 1.355]	1.000 [0.730, 1.445]	

Analysis was performed on the first imputed dataset. Continuous variables are presented as median [interquartile range] and were compared using the Mann-Whitney U test. Categorical variables are presented as n (%) and were compared using the Pearson chi-square test or Fisher’s exact test (when any expected cell count was <5). All tests were two-tailed, and a P-value < 0.05 was considered statistically significant. This table does not adjust for multiple comparisons, as it is intended for descriptive purposes only. Hemoglobin (Hb) was sex-standardized and is presented as mean (SD). Ratio variables (LMR, NLR, PLR, NPR, PAR) are unitless.

Laboratory findings indicated that patients in the PHN group had more significant inflammatory activation and metabolic disturbances. Specifically, serum albumin levels were significantly lower in the PHN group than in the control group (*p* < 0.001). Regarding inflammatory markers, ALC (*p* = 0.041) and LMR (*p* = 0.048) were significantly lower in the PHN group, while CRP was significantly higher (*p* < 0.001). In addition, HbA1c levels were also significantly higher in the PHN group than in the control group (*p* < 0.001). No significant differences were found between the two groups for other cell counts and derived ratios, such as NLR and PLR.

### 3.2 Confounding factor adjustment

To control for potential confounding factors, a multivariable logistic regression analysis was performed adjusting for age, gender, diabetes history, and CCI score (see **Table 2**). The results showed that age and gender were independently associated with PHN (all *p* < 0.05). In contrast, the other variable did not reach statistical significance after adjustment for confounders, suggesting that the associations of these indicators with PHN may exhibit nonlinear characteristics.

**Table 2:** Confounding factor adjustment (Multivariable logistic regression analysis for PHN risk).

Variable	OR (95% CI)	*P* value2
Age	1.04 (1.02–1.06)	<0.001
Gender (Male vs Female)	0.63 (0.40–1.00)	0.049
Diabetes_History (Yes vs No)	1.02 (0.53–1.97)	0.95
HbA1c	1.20 (0.93–1.55)	0.16
CCI_Score	1.24 (0.77–1.99)	0.38
Cancer_History (Yes vs No)	0.47 (0.18–1.17)	0.11

Multivariable logistic regression was adjusted for six confounders (Age, Gender, Diabetes_History, HbA1c, CCI_Score, Cancer_History), based on 10 imputed datasets and pooled using Rubin’s rules. Reference categories: female for Gender, no for Diabetes_History, and no for Cancer_History.

### 3.3 Integrated feature screening process

#### 3.3.1 Results of integrated feature screening

The results of the three independent feature screening methods are as follows:

RF: The top 20 features in terms of importance are shown in **Supplementary Figure 2A**, with age, NLR, and ALB being the most significant.

LASSO regression: Using the lambda.1se criterion (λ = 0.1039), 21 predictors with non-zero coefficients were selected from the 29 initial variables. The ranking of the standardized coefficients (absolute values) for each variable is shown in **Supplementary Figure 2B**.

Boruta algorithm: After comparison with shadow features, 4 features were confirmed as “important” and 7 features were classified as “tentative”, as shown in **Supplementary Figure 2C**.

By applying min-max normalization and equal-weight integration to the three methods described above, a comprehensive importance score was calculated for each feature, and the features were ranked accordingly. The results are presented in **Supplementary Figure 2D**.

#### 3.3.2 Determination of the optimal feature number and final feature set

**Figure 2:**
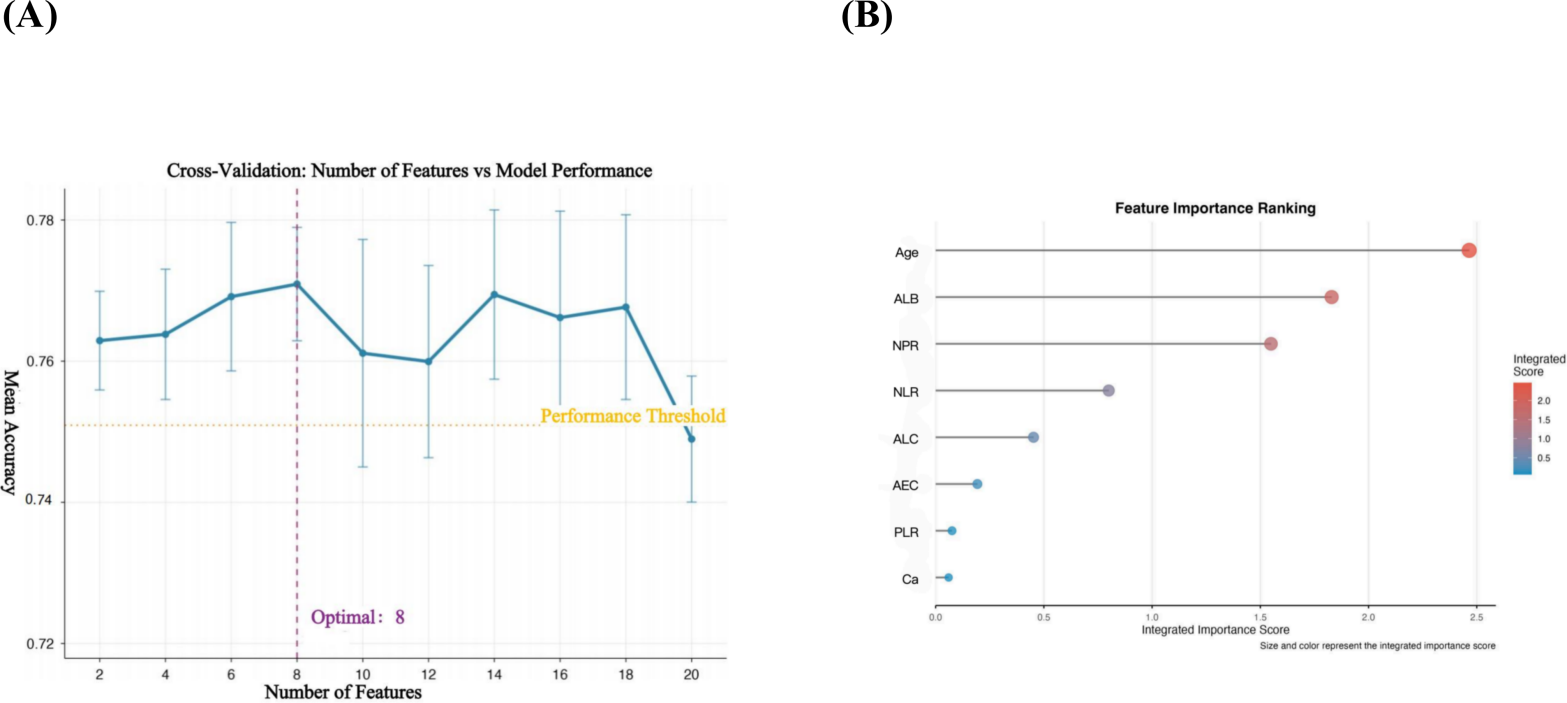
Optimal feature selection **(A)** Cross-validation of model performance changes based on the number of selected features. The purple dashed line indicates the number of feature subsets that enables the model to achieve optimal performance. **(B)** The final selected 8 predictive features and their ranking based on the integrated score.

Based on the composite importance score, a logistic regression model was employed to evaluate the cross-validation performance with different numbers of top-ranked features. As shown in **Figure 2A**, the average accuracy peaked when the number of features was eight. A performance plateau analysis indicated that, within a tolerance of no more than a 2% decrease in accuracy, the minimum feasible number of features was also eight. When the number of features exceeded eight, the accuracy plateaued or even declined (*p* > 0.05 for the improvement beyond eight features), suggesting a potential risk of overfitting. Therefore, eight features were selected to minimize model complexity while maintaining optimal performance. The final set of eight key predictors, listed in descending order of their comprehensive importance, were: age, ALB, NPR, NLR, ALC, AEC, PLR, and Ca (see **Figure 2B**).

Feature stability and weight sensitivity analyses: to assess the robustness of the feature selection, two complementary stability analyses were performed (**Supplementary Table 6**). Age and ALB exhibited “extremely high” stability (selection frequency ≥ 0.91) under both random partitioning and bootstrap resampling, indicating that they are the core pillars of the model. ALC and AEC showed “high” to “moderate” stability, whereas NLR, Ca, NPR, and PLR showed “moderate” to “low” stability, which is consistent with their nonlinear, interaction-driven roles.

In the weight sensitivity analysis, when the weight of any single method was doubled (i.e., weight ratios of 2:1:1, 1:2:1, or 1:1:2), the Jaccard similarity coefficient between the selected feature set and the equal-weight scheme remained ≥ 0.778. In contrast, when only a single method was used, the Jaccard coefficient dropped to as low as 0.143 (for LASSO alone), confirming the necessity of integrating the three methods and the robustness of the equal-weight approach (**Supplementary Table 7**).

### 3.4 Performance comparison of machine learning models

Based on the selected eight key predictors, the performance of six machine learning models (XGBoost, SVM, RF, NN, LightGBM, and LR) was systematically evaluated on the test set using nested cross-validation. The evaluation covered discrimination, calibration, clinical utility, and overall performance.

#### 3.4.1 Discriminatory performance

**Figure 3:**
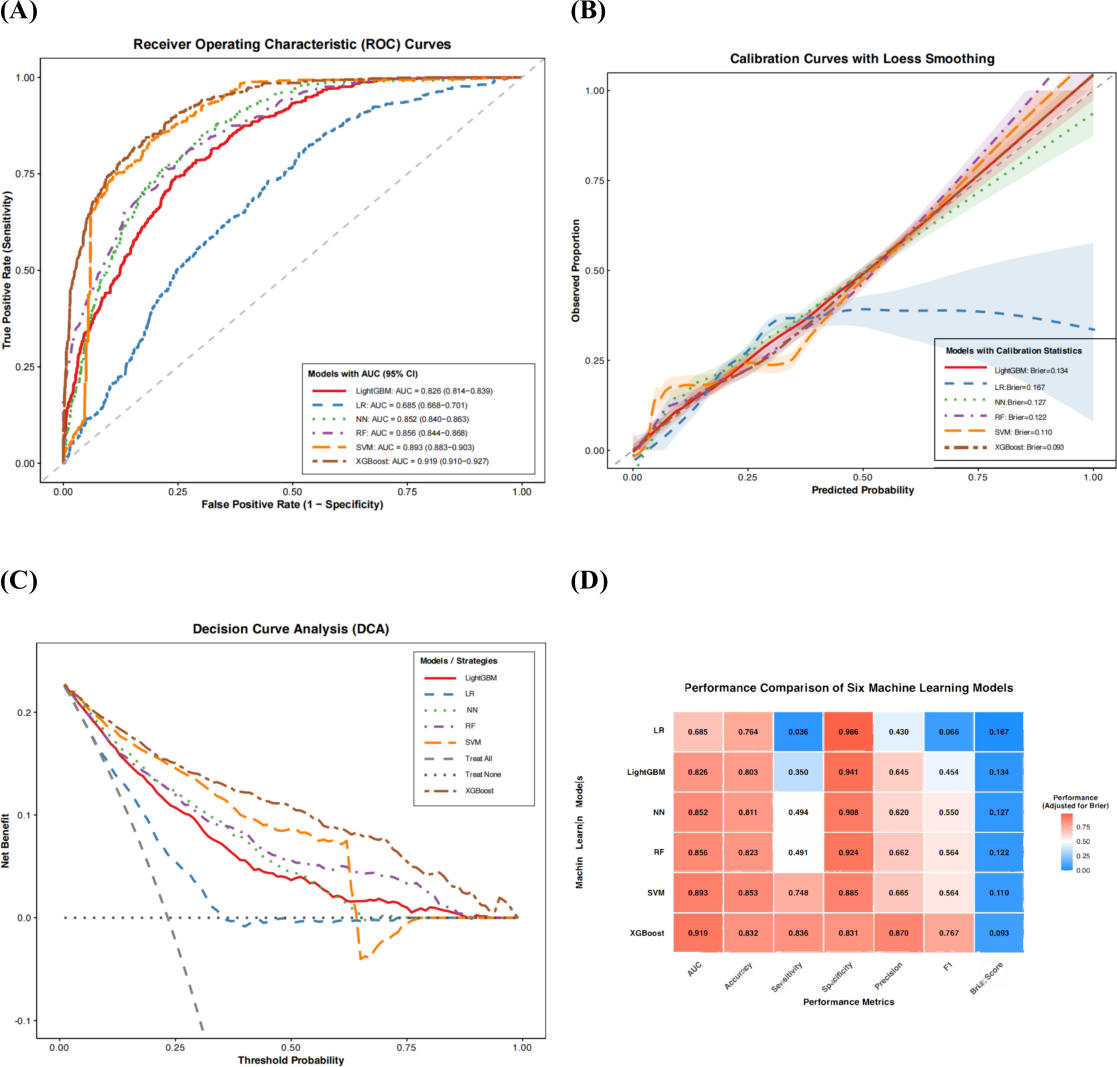
Performance comparison of machine learning models **(A)** Receiver operating characteristic curve. Light GBM, light gradient boosting machine; LR, Logistic regression; NN, neural network; RF, random forest; SVM, support vector machine; XG Boost, extreme gradient boosting. **(B)** Calibration curve for predicted probabilities. **(C)** Clinical decision curve analysis. **(D)** Heatmap of classification performance metrics.

The ROC curves for each model are shown in **Figure 3A**. The XGBoost model demonstrated the best discriminatory performance, achieving an area under the curve (AUC) of 0.919 (95% CI: 0.910–0.927). The AUC values of the remaining models, in descending order, were: SVM (0.893, 95% CI: 0.883–0.903), RF (0.856, 95% CI: 0.844–0.868), NN (0.852, 95% CI: 0.840–0.863), LightGBM (0.826, 95% CI: 0.814–0.839), and LR (0.685, 95% CI: 0.668–0.701).

#### 3.4.2 Calibration performance

The calibration curves for each model (calibrated using the Platt scaling method) are shown in **Figure 3B**. The XGBoost model exhibited favorable calibration, with its calibration curve lying closest to the diagonal (the ideal reference line). All models had calibration slopes greater than 0.85, indicating that the predicted risks were generally accurate and reliable.

#### 3.4.3 Clinical utility

Decision curve analysis (**Figure 3C**) was used to evaluate the net clinical benefit of each model across different threshold probabilities. Within the clinically relevant threshold range of 10% to 50%, all models except LR demonstrated a higher net benefit than the **“**treat-all**”** and **“**treat-none**”** strategies. The XGBoost model provided the highest net benefit over the entire threshold range.

#### 3.4.4 Overall performance comparison

The heatmap in **Figure 3D** provides a comprehensive comparison of the six models across multiple performance metrics, including Brier score, accuracy, sensitivity, specificity, and F1 score. The XGBoost model achieved the best performance on most of these metrics. In contrast, the overall performance of the LR model was relatively limited. This comprehensive assessment further confirms that the ensemble-based XGBoost model is optimal for the prediction task in this study.

### 3.5 Performance and interpretability analysis of the optimal model (XGBoost)

#### 3.5.1 Model performance and SHAP global interpretation

**Figure 4:**
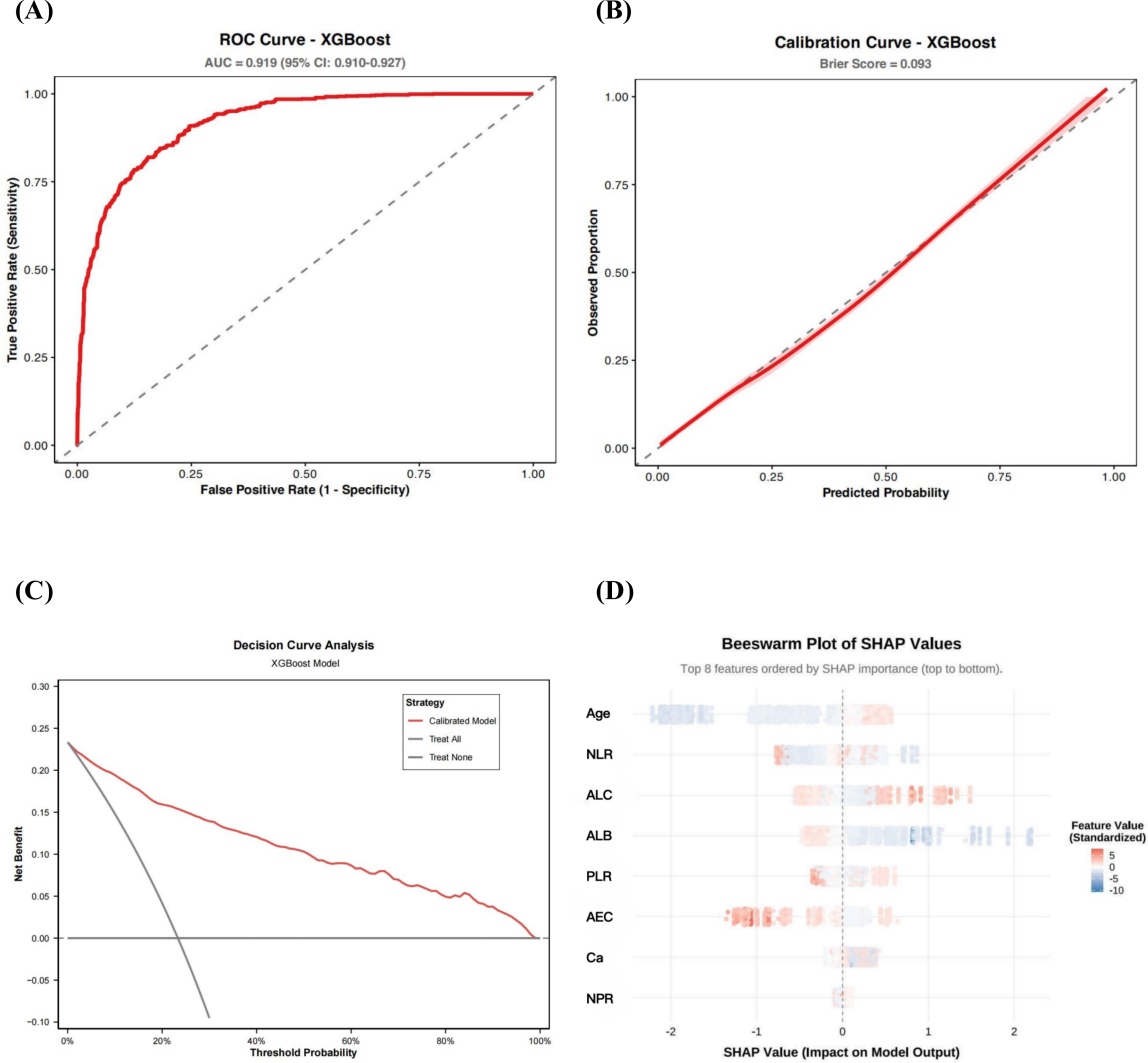
Performance and interpretability analysis of the optimal model (XGBoost) **(A)** Receiver operating characteristic curve. **(B)** Calibration curve for predicted probabilities. **(C)** Clinical decision curve analysis. **(D)** Beeswarm plot.

After a comprehensive comparison, XGBoost was determined to be the optimal prediction model. Its performance on the test set is shown in **Figure 4**. **Figure 4A** presents the ROC curve, demonstrating its strong discriminatory ability with an AUC of 0.919 (95% CI: 0.910–0.927). Detailed performance metrics, including accuracy, sensitivity, specificity, F1 score, and Brier score, are summarized in **Table 3**. **Figure 4B** shows the calibration curve, indicating excellent agreement between the predicted probabilities and the actual observed outcomes. **Figure 4C** displays the decision curve analysis, which confirms that the model provides a higher net benefit across a wide range of clinically reasonable threshold probabilities (detailed net benefit values at key thresholds are provided in **Supplementary Table 8**).

**Table 3:** Results and confidence intervals for key metrics.

Metric	Point estimate	95% confidence interval
AUC	0.919	(0.910 – 0.927)
Accuracy	0.832	(0.821 – 0.842)
Sensitivity	0.836	(0.757 – 0.903)
Specificity	0.831	(0.789 – 0.869)
Positive Predictive Value (PPV)	0.767	(0.685 – 0.836)
Negative Predictive Value (NPV)	0.943	(0.914 – 0.967)
Youden’s Index	0.667	(0.644 – 0.693)
Brier Score	0.093	(0.081 – 0.106)

To interpret the model, SHAP analysis was performed. **Figure 4D** is a SHAP summary (beeswarm) plot based on mean absolute SHAP values, with features listed in descending order of importance: age, NLR, ALC, ALB, PLR, AEC, Ca, and NPR. The plot also reveals the preliminary directional effects of each feature on PHN prediction: The SHAP values for age are entirely distributed to the right of the zero axis, indicating a consistently positive contribution to the model output. The SHAP values for NLR, PLR, and Ca are distributed on both sides of the zero axis, suggesting a nonlinear or complex association with PHN risk. The data points for ALB, ALC and AEC are predominantly clustered on the left side of the zero axis, indicating a predominantly negative effect. The data points for NPR show a relatively narrow and dispersed distribution, suggesting that its overall influence in the current model is relatively limited.

#### 3.5.2 SHAP dependency analysis

**Figure 5:**
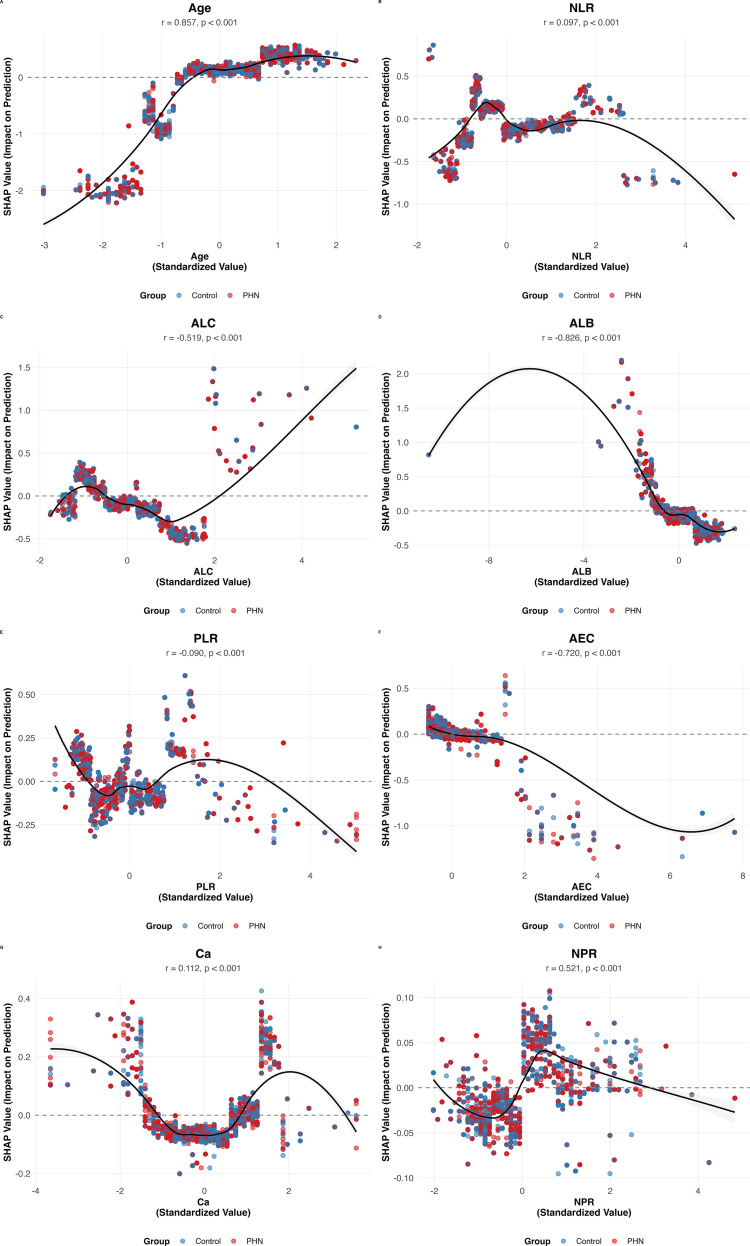
SHAP dependence plot Eight individual panels display the relationship between each standardized clinical feature (x-axis) and its SHAP value (y-axis), which quantifies the feature’s contribution to the model’s predicted PHN risk. Positive SHAP values increase the predicted probability of PHN, while negative values decrease it. Points are colored by patient group: blue for control subjects, red for PHN patients. The black solid line represents the LOESS (locally estimated scatterplot smoothing) trend with a 95% confidence band (grey shading), revealing the overall functional relationship. The dashed horizontal line at y = 0 serves as a reference for zero contribution. The Spearman correlation coefficient (*r*) between each feature and its SHAP value is displayed in each subplot, with significance indicated as *p* < 0.001, *p* < 0.01, *p* < 0.05. Positive *r* indicates that higher feature values are associated with increased PHN risk, while negative *r* indicates the opposite. Non-linear patterns reveal threshold effects or complex risk relationships where the direction or magnitude of association changes across the feature’s range.

To further elucidate the specific contribution patterns of key features, we generated SHAP dependence plots, as shown in **Figure 5**, revealing the estimated marginal effects of each feature within the optimal model. It should be noted that these plots illustrate statistical associations identified by the model and do not imply causal relationships. Age exhibited a stable, monotonically increasing positive effect, indicating a robust linear predictor of higher PHN risk(*r* = 0.8568). NLR, PLR, and Ca each showed a U-shaped nonlinear relationship with PHN risk. The Spearman’s correlation coefficients were *r* = 0.00971 for NLR, *r* = −0.0902 for PLR, and *r* = 0.1120 for Ca. ALB, ALC, and AEC demonstrated a linear negative correlation, with lower levels associated with higher PHN risk (*r* = −0.8259, *r* = −0.5194, and *r* = −0.7198, respectively). NPR showed a positive correlation with PHN risk.

#### 3.5.3 Interaction analysis

To explore the potential complex synergistic relationships among key predictors, pairwise interactions between all biomarkers were systematically evaluated (detailed results are provided in **Supplementary Tables 9**, **10**; **Supplementary Figure 3**). The results suggested the presence of a potential risk-modification network. It should be noted that SHAP interaction analysis is a visual exploratory tool; its findings are not intended for statistical inference and should be used only to generate hypotheses for future research.

##### 3.5.3.1 Synergistic effect patterns based on SHAP interaction values

The interaction table quantifies the net effect attributable solely to the co-variation of two features after removing their independent main effects. Among the 28 pairs, 24 exhibited significant interactions after Bonferroni correction (adjusted *p* < 0.0018). The remaining four pairs (Age–NPR, Age–AEC, NLR–Ca, and PLR–Ca) did not reach the corrected significance level and were not interpreted in detail. Four main synergistic patterns were identified.

First, synergistic risk reduction: When two features were simultaneously at high levels, their combined effect produced an additional risk-reducing effect. The interaction between age and ALB was the strongest (Max SHAP Diff = 0.437) and most significant (*p* = 7.43 × 10^-94^), with their synergy reducing risk when both were high (SHAP interaction value = -0.382). Similar synergistic risk-reduction patterns were observed between age and ALC, and between age and Ca (SHAP interaction value = -0.295 and -0.254, respectively). These findings suggest that protective factors (high ALB, high ALC) are synergistically amplified in elderly patients, implying greater benefits from maintaining good nutritional and immune status in older individuals.

Second, synergistic risk increase and nonlinear reversal: The interaction between ALB and Ca significantly increased risk when both were low (SHAP interaction value = 0.122). The interaction between ALC and PLR exhibited a critical nonlinear reversal: It reduced risk when both were at low or medium level, but reversed to a slight risk increase when both were high (SHAP interaction value = 0.051). This reversal, despite its modest magnitude, had high statistical certainty (*p* = 2.66 × 10^-223^), suggesting the possible existence of a qualitative threshold in the immune-inflammatory balance.

Third, systematic modulatory role of AEC: AEC showed consistent “low-positive, high-negative” patterns when interacting with ALB, NLR, and Ca (slightly increasing risk when both were low, synergistically reducing risk when both were high). This indicates that the protective role of AEC is enhanced under a favorable systemic state.

Fourth, the dominant NLR–ALC axis: The interaction between NLR and ALC was highly significant (F = 419.6, *p* < 0.001), demonstrating that the risk implication of NLR fundamentally depends on the lymphocyte background. This pair, along with the reversal patterns observed for NLR with AEC and PLR (see below), positions the NLR–ALC interaction as a central regulatory hub.

##### 3.5.3.2 Visualization of complex relationships based on interaction dependency plots

Interaction dependency plots illustrate how the total contribution (SHAP value) of one feature is modified by its own value and the level of another feature (the conditioning variable). We calculated the Spearman correlation coefficient (r) for each interaction pair at three tertiles of the conditioning feature and defined Δr as the difference between the maximum and minimum r values to measure interaction strength.

NLR–ALC (Δr = 0.851, strongest). When ALC was low, NLR showed a negative correlation (r = –0.259); when ALC was high, the correlation turned positive (r = 0.592). This reversal indicates that the inflammatory risk direction depends critically on the immune background. Similar direction reversals were observed for NLR–AEC (Δr = 0.788) and NLR–PLR (Δr = 0.748), suggesting a general conditional dependence of inflammatory markers.

ALC and PLR showed a nonlinear modulating pattern. ALC consistently exhibited a protective effect (negative correlation), but the strength of this protection varied with PLR levels: strongest at medium PLR (r = –0.763) and weaker at high PLR (r = –0.279).

Age displayed additive effects in its interactions with NLR, ALC, and AEC. The stratified correlation coefficients for age were highly stable (r range 0.83–0.87, Δr < 0.04), indicating that age acts independently of immune-inflammatory markers and can be used in additive risk assessment.

##### 3.5.3.3 Integrated framework

Integrating dependency plots, interaction tables, and interaction graphs, the PHN risk prediction network is dominated by the NLR–ALC axis (direction reversal, highly statistically significant). Age serves as an independent baseline factor, while ALB, ALC, and AEC act as modifiable protective regulatory factors. Together, these findings suggest that PHN risk is a complex phenotype regulated by an inflammatory-immune network, rather than a linear function of any single feature.

#### 3.5.4 Model robustness (Sensitivity) analysis and clinical tool development

To evaluate the robustness and clinical applicability of the finalized XGBoost model, a systematic sensitivity analysis was performed, and an interactive clinical prediction tool was developed based on the model.

##### 3.5.4.1 Complete-case sensitivity analysis

To validate the necessity of multiple imputation, a complete-case sensitivity analysis was conducted (1). Missing mechanism analysis: Among the 480 full samples, 415 cases (86.5%) had complete data for the eight core variables. Analysis of the outcome distribution in the 65 cases with missing data showed that 58 cases (15.8% of the control group) were excluded due to missingness on at least one core variable, whereas only 7 cases (6.2% of the PHN group) were excluded. The difference in missing proportions between groups was statistically significant (Wilcoxon test, *p* = 0.0093). This finding indicated that the missing data were not missing completely at random (MCAR) but were associated with the outcome variable (PHN status), consistent with missing at random (MAR) or missing not at random (MNAR) mechanisms. Direct use of complete-case analysis would disproportionately exclude information from the control group and potentially introduce selection bias (**Supplementary Table 11**).

(2) Model performance comparison: On the complete-case subset (n = 415), the XGBoost model achieved an AUC of 0.657 ± 0.051, accuracy of 0.636 ± 0.070, sensitivity of 0.562 ± 0.103, and specificity of 0.661 ± 0.081 using nested cross-validation. In contrast, the primary analysis (after multiple imputation; n = 480) yielded an AUC of 0.919 (95% CI: 0.910–0.927), an accuracy of 0.874, a sensitivity of 0.836, and a specificity of 0.831. The complete-case analysis resulted in a reduction in AUC of approximately 0.262 compared with the primary analysis, and other performance metrics also being substantially inferior to those of the primary analysis (**Supplementary Table 12**).

(3) Feature importance changes: In the complete-case analysis, the ranking of feature importance was age, ALB, ALC, and NLR. In the primary analysis, however, the relative importance of inflammatory and immune markers such as NLR was substantially higher. This change indicated that the complete-case analysis, due to compromised sample representativeness, may have underestimated the contribution of inflammatory and immune markers to PHN risk prediction.

Conclusion: This sensitivity analysis confirms that, because missingness is associated with the outcome variable, complete-case analysis introduces selection bias and leads to a marked decrease in model performance. Multiple imputation preserves full sample information, effectively restores sample representativeness, and enables the model to achieve excellent discriminative performance. Therefore, the use of multiple imputation in the primary analysis was necessary and justified.

##### 3.5.4.2 Stability of core predictor contributions

**Figure 6:**
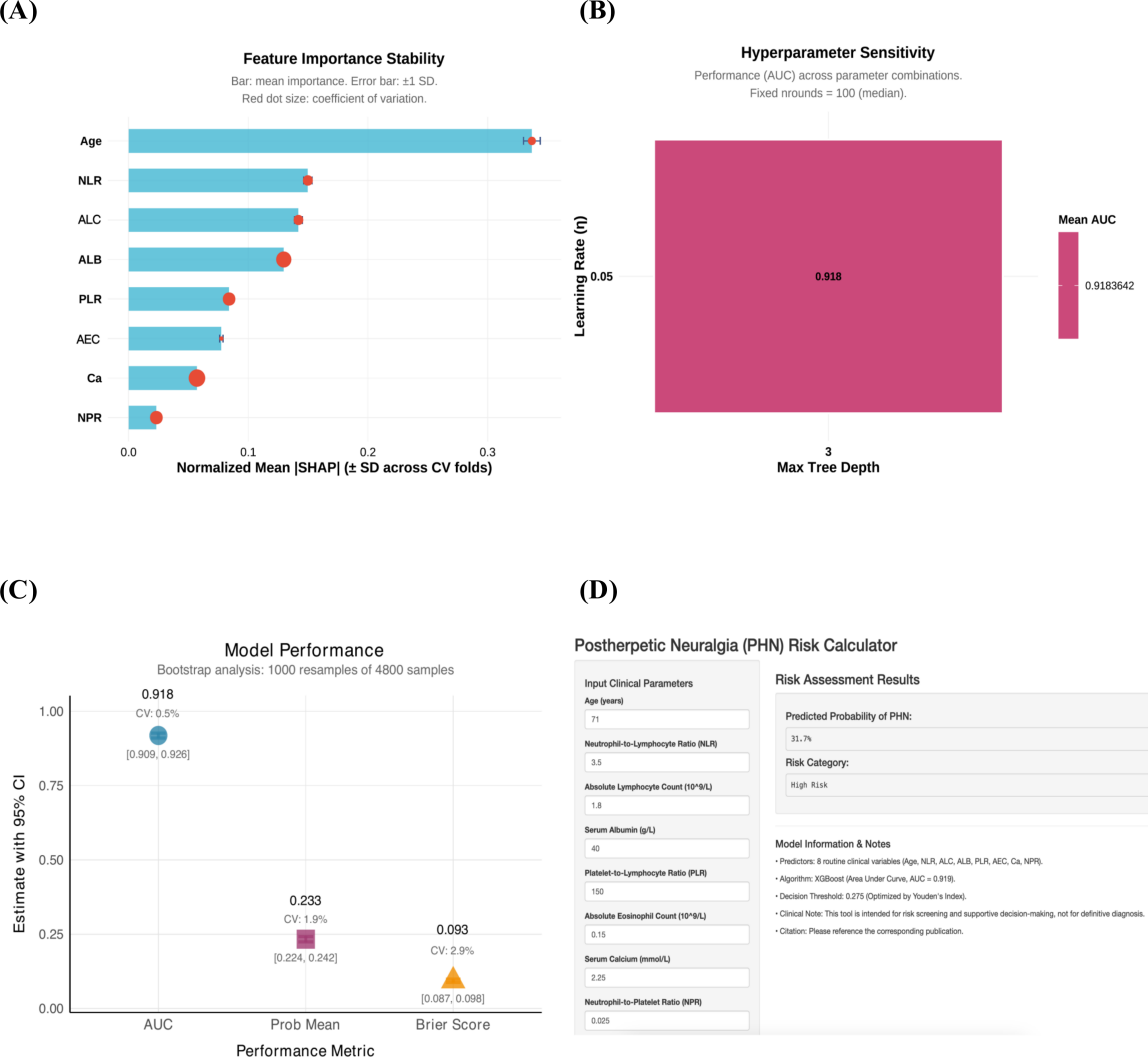
Model robustness (Sensitivity) analysis and online risk calculator **(A)** Feature importance stability. **(B)** Hyperparameter sensitivity. **(C)** Bootstrap internal validation and performance consistency. **(D)** Online risk calculator.

SHAP feature importance was recalculated across 100 random data splits. As shown in **Figure 6A**, age, NLR, and ALC were the most influential and stable predictors, with their importance rankings being highly consistent across iterations. The coefficient of variation (CV) for each feature’s importance was generally low (median: 0.026). Among them, the contribution of age was the most stable (relative importance: 33.7%, CV = 0.021). In contrast, the importance of features such as ALB and NPR showed relatively greater variability; however, the overall CV was below 5%, indicating high model robustness and reproducibility (see **Supplementary Table 13** for the variation values of each feature).

##### 3.5.4.3 Hyperparameter sensitivity

The sensitivity of model performance to two key hyperparameters is shown in **Figure 6B**. The results indicated that performance was most sensitive to the learning rate: lower settings (0.01–0.05) were associated with better AUC. In contrast, the maximum tree depth had a relatively limited impact on final performance within the tested range (3–10), indicating that the model was insensitive to this parameter and structurally robust.

##### 3.5.4.4 Bootstrap internal validation and performance consistency

To provide a reliable interval estimate of the model’s discriminative performance, we performed internal validation using 1,000 Bootstrap resamples. The model demonstrated high performance stability, with a mean AUC of 0.910 (95% CI: 0.859–0.951). This result was highly consistent with the optimal AUC obtained from the rigorous 5 × 5 nested cross-validation (0.919) and the optimal AUC from the hyperparameter tuning process (0.918), with minimal absolute difference (< 0.9%). The nested cross-validation AUC fell entirely within the 95% confidence interval of the Bootstrap estimate (**Figure 6C**), further supporting the robustness of the model performance estimate from multiple perspectives.

### 3.6 Development and presentation of an interactive web calculator

Based on the calibrated XGBoost model, a publicly accessible interactive web calculator was developed (**Figure 6D**; available at: https://kangpeipei.shinyapps.io/phn_web). The system returns a personalized PHN risk probability for the patient in real time. To determine the optimal decision threshold, the Youden index was applied, which balances sensitivity and specificity. The maximum Youden index was 0.667 (95% CI: 0.644–0.693), corresponding to an optimal threshold of 27.5% (95% CI: 22.3%–31.5%). At this threshold, the model achieved a sensitivity of 83.6% and a specificity of 83.1%. A predicted probability ≥ 27.5% is classified as high risk for PHN, suggesting close follow-up or evaluation of preventive intervention in conjunction with clinical features; a probability < 27.5% is considered low risk. The model was characterized by a high negative predictive value (94.3%, 95% CI: 91.4%–96.7%) and a moderate positive predictive value (76.7%, 95% CI: 68.5%–83.6%). Declaration: The online calculator is currently provided as a research prototype. Its clinical utility requires confirmation through independent external validation and prospective impact assessment before clinical translation can be considered.

## 4 Discussion

This study developed and internally validated a machine learning model for predicting PHN risk based on single-center retrospective data. By integrating multiple imputation, comprehensive feature engineering, and a nested cross-validation framework comparing six algorithms, XGBoost was identified as the optimal model. The model showed favorable discriminatory ability and clinical utility in internal validation, providing an exploratory tool for risk stratification and individualized prevention of PHN. It should be emphasized that the following discussion of biological mechanisms is based on statistical associations derived from SHAP analysis, representing data-driven hypothesis generation rather than causal conclusions.

### 4.1 Rationale for multiple imputation and feature selection robustness

Multiple imputation was necessary because missingness was associated with the PHN outcome; complete-case analysis led to substantially reduced model performance and underestimated the contribution of inflammatory markers. Nevertheless, imputation cannot completely eliminate bias if missingness depends on unmeasured factors (MNAR). The integrated feature selection strategy (Boruta, random forest, and LASSO) proved robust, with weight sensitivity analysis confirming its superiority over any single method. XGBoost significantly outperformed logistic regression, suggesting that PHN pathogenesis may involve complex nonlinear interactions among variables. However, due to the single-center retrospective design and limited sample size, all inferences regarding generalizability require caution, and external validation is necessary.

### 4.2 Integrative biological interpretation of key predictors: from linear associations to a networked imbalance

SHAP analysis not only identified key predictors but also revealed, through interaction analysis, that these factors may not act in isolation but rather constitute a dynamic interaction network, offering a novel integrative perspective for understanding the heterogeneity of PHN.

#### 4.2.1 Aging and nutritional metabolism: the stable basis and modifiability of risk

Age was identified as the most important predictor, consistent with previous studies showing that immunosenescence, declined neural repair capacity, and an increased comorbidity burden associated with advanced age collectively elevate the risk of PHN (16). ALB is a key molecule for maintaining homeostasis, and its independent protective effect aligns with prior research (28) and is supported by cross-disciplinary evidence (29, 30). A novel finding of this study is the strong synergistic protective effect observed between age and ALB, suggesting that good nutritional status may buffer the accumulation of PHN risk attributable to aging. This provides a theoretical basis for nutritional intervention in elderly patients with HZ. Furthermore, ALB also exhibits strong interactions with Ca and AEC, suggesting that its nutritional and immunomodulatory functions are embedded within a broader physiological network.

#### 4.2.2 The immune-inflammatory network: conditional dependence and dynamic Balance

Peripheral blood indicators, particularly NLR, PLR, AEC, and ALC, collectively reflect the body’s inflammatory status, nutritional state, and basal immune competence and are widely used for diagnosis, prognosis, prediction, treatment monitoring, and drug response of various diseases (19, 21, 27, 28, 31–35). This study not only confirms the key predictive role of these indicators in PHN but also demonstrates through interaction analysis that these key indicators do not act in isolation; instead, they constitute a dynamic, condition-dependent network.

##### 4.2.2.1 Lymphocytes: key regulators with context-dependent roles

Lymphocytes play a pivotal role in regulating the immune response against the varicella-zoster virus (VZV). Studies have shown that an imbalance in peripheral blood T-cell subsets during the acute phase of HZ can significantly hinder viral clearance and neural repair (36). Furthermore, lymphocytes exhibit a biphasic dynamic during inflammation (37): they are cleared in the initial phase, and as inflammation subsides, they repopulate the injury site with a phenotype distinct from their initial state, suggesting that the intensity, timing, and resolution order of the immune response collectively influence disease outcomes. Although univariate analysis confirmed the association between lymphopenia and PHN, our model further identified a subset of individuals with high ALC levels who were also at high risk. Interaction analysis revealed a nonlinear reversal pattern between ALC and PLR. Multiple independent studies (39–41), including CyTOF, Mendelian randomization, and flow cytometry analyses, have provided supportive evidence. These findings are consistent with the core hypothesis generated by our model: It is the dynamic balance of immune function, rather than the absolute increase or decrease in ALC, that is central to understanding the pathogenesis of PHN.

##### 4.2.2.2 NLR and PLR: composite markers reflecting systemic inflammation

Both NLR and PLR are established composite indices of systemic inflammation. Consistent with prior research (19, 21), NLR and PLR were found to be important predictors with complex roles. A high NLR coexisting with high ALC or AEC levels may produce a synergistic protective effect. Conversely, a low NLR combined with lymphopenia or eosinopenia may amplify risk, potentially reflecting an ineffective or exhausted immune state. This indicates that the harm of systemic inflammation is not absolute; rather, the key lies in whether it is balanced by an effective adaptive (ALC) or regulatory (AEC) immune response. In contrast to previous studies, our study found that PLR is highly nonlinear, and its risk implication partially depends on the levels of ALC and AEC. This further suggests that the interpretation of PLR cannot be separated from an assessment of overall immune cell functional status.

##### 4.2.2.3 Eosinophils: key immunomodulators and homeostatic contributors

Eosinophils are multifunctional granulocytes involved not only in antiparasitic and allergic reactions but also in immune regulation, metabolism, and tissue repair, acting as contributors to homeostatic processes (38, 42). They can shape the overall immune response through antigen presentation, cytokine secretion, and intercellular crosstalk (43). Although eosinophils have not been a primary focus in HZ and PHN research, in oncology, an elevated AEC is associated with a favorable prognosis and improved response to immunotherapy (44, 45). In infectious diseases, a low AEC is significantly linked to worse clinical outcomes and heightened inflammation (42, 46–48). This cross-disciplinary evidence collectively points to AEC as a crucial node for maintaining immune homeostasis and promoting orderly inflammation resolution. A novel finding of this study is the observed inverse association between AEC and PHN risk, which has not been reported previously in PHN prediction models. Furthermore, interaction analysis revealed that interactions between AEC and ALB, NLR, and Ca all exhibited a consistent “low-positive, high-negative” synergistic pattern. This further suggests that AEC may serve as a bridge linking systemic physiological status with the local immune microenvironment, and that its functional integrity may help mitigate the positive contribution of other inflammatory or immune indicators to PHN risk. However, this finding is exploratory, and the specific mechanisms require experimental validation.

##### 4.2.2.4 NPR: An emerging marker integrating acute inflammation and chronic consumption

NPR is an emerging inflammatory marker that integrates signals of acute inflammatory activation and a chronic consumptive state (49, 50). Previous studies have confirmed the value of NPR in conditions like infective endocarditis and in predicting multidrug-resistant urinary tract infections in patients with brain and spinal cord injuries (51, 52). This study also observed an association between a high NPR and increased PHN risk, which may suggest the involvement of inflammation-microcirculation disorders in suboptimal neural repair following injury. Its relatively weak effect within the interaction network suggests that NPR may be more indicative of the severity of acute inflammation and systemic consumption rather than the nuanced state of immune regulation.

#### 4.2.3 Ca: a key metabolic and signaling molecule

Ca is a key metabolic and signaling molecule whose homeostasis influences both neuronal excitability and immune regulation (53). This study found a nonlinear association between Ca level and PHN risk, along with significant synergistic effects with indicators such as ALB and AEC. This suggests that the impact of Ca on PHN risk is more likely to reflect “an imbalance in calcium homeostasis,” and that this effect is realized by being embedded within a systemic network formed by nutritional status (ALB) and immunomodulation (AEC). Therefore, Ca should be interpreted within a broader metabolic and immune context during clinical assessment.

In summary, the high performance of this model stems from the simultaneous integration of strong linear predictors and immune-inflammatory indicators with complex nonlinear relationships. Interaction analysis suggests that PHN risk is determined by a complex, networked imbalance among systemic inflammation, cellular immune response efficacy, and intrinsic immunoregulatory function, all operating on a foundation of aging and nutritional metabolism. All of these interpretations are based on exploratory hypotheses derived from retrospective data and require validation in independent prospective cohorts and mechanistic studies.

### 4.3 Comparison with existing research and innovations

This study systematically reviewed eight representative PHN prediction models (see **Supplementary Tables 14A, B**). Our XGBoost model (AUC: 0.919) outperformed all previous models with the exception of one study that reported an AUC range of 0.87–0.907 (54). Compared with previous studies, our model offers three major advantages:

First, more rigorous methodology. Most previous studies employed a single screening strategy. In contrast, our study integrated three complementary feature selection methods, which enhanced the robustness of predictor identification. Furthermore, the use of nested cross-validation provides a less biased performance estimate than conventional split-sample or standard cross-validation. Notably, one previous study (55) employed external validation, which is more rigorous than internal validation; however, our model achieved a competitive AUC (0.919 vs. 0.84) in an internal setting, suggesting potential for further external validation.

Second, more objective predictors with greater clinical accessibility. Most previous models (36, 56) relied on non-routine indicators, subjective scores (21, 54), or expensive tests (54, 56), limiting their clinical applicability. Our model requires only eight routine laboratory indicators (complete blood count and biochemistry), does not depend on any subjective scores, and is feasible across all levels of healthcare settings.

Third, stronger model interpretability. While most previous studies only reported predictive performance, our model employed SHAP analysis to reveal nonlinear interactions among variables, providing a preliminary outline of a potential risk-modification network involving the “immune-inflammation-aging” axis and generating new hypotheses for understanding PHN pathogenesis.

In summary, our model offers distinct advantages in methodological rigor, clinical accessibility, and model interpretability. Nonetheless, the findings presented here are subject to several limitations, which are addressed in detail in the Limitations section.

### 4.4 Limitations

Several limitations of this study should be acknowledged.

First, inherent bias of the single-center retrospective design. Although patients were consecutively enrolled, the incidence of PHN in hospitalized patients (23.3%) was higher than that reported in a nationwide cross-sectional study (15.8%), suggesting potential selection bias. Unmeasured confounders (e.g., detailed immunophenotypes, genetic background) cannot be ruled out. As a result, the generalizability and clinical credibility of the model may be compromised.

Second, insufficient statistical power for detecting complex interactions. The sample size of 480 is relatively modest for the initial 39 features and subsequent higher-order nonlinear interaction analyses. Although nested cross-validation, integrated feature selection, and bootstrap stability testing were employed, these cannot fully compensate for the inherent limitation of sample size. All interaction findings should therefore be considered exploratory hypotheses that require validation in independent large-scale cohorts.

Third, risk of outcome misclassification due to non-structured information. Standardized neuropathic pain scales (e.g., ID Pain, DN4) were not used, and pain intensity was not quantitatively recorded using VAS or NRS. The follow-up time point was not strictly uniform. Recall bias and information misclassification are possible and represent inherent limitations of this retrospective design.

Fourth, uncertainty in missing data handling. Although multiple imputation led to markedly better model performance than complete-case analysis, and the missing mechanism analysis suggested an association between missingness and the outcome, the possibility of missing not at random (MNAR) cannot be completely excluded. This constitutes an inherent uncertainty in missing data processing.

Fifth, methodological dependence of feature selection stability. Stability analysis showed that age and ALB had extremely high stability, whereas NLR, PLR, and Ca exhibited lower selection frequencies, indicating that the selection of these predictors is sensitive to the training set composition, which is consistent with their nonlinear, interaction-driven nature.

Sixth, lack of external validation and impact assessment. This study did not perform any external or prospective validation, nor did it conduct a formal impact analysis (e.g., cluster randomized controlled trial). The online calculator is currently a research prototype, and its real-world clinical utility requires independent validation before clinical application can be considered.

Seventh, risk of over-interpretation of mechanisms. The nonlinear relationships and interactions revealed by SHAP analysis are statistical associations, not causal effects. All biological interpretations are exploratory hypotheses and should not be misinterpreted as established pathological mechanisms.

Eighth, omission of important clinical features due to data quality issues. Clinically relevant variables such as rash area and the nature of prodromal pain could not be included because of incomplete or non-standardized documentation in the medical records, limiting the completeness of the model.

### 4.5 Conclusions and future directions

Based on single-center retrospective data, this study developed and internally validated an XGBoost model with favorable performance and clinical interpretability for predicting PHN risk. The model not only confirmed key risk factors but also, through interaction analysis, suggested the presence of a networked imbalance involving multiple systems (immune, inflammatory, and nutritional) underlying PHN occurrence. This provides an integrative exploratory framework that goes beyond single biomarkers for understanding the heterogeneous mechanisms of PHN and lays a hypothesis-generating foundation for developing precision prevention strategies targeting the “network imbalance.”.

Future directions include: conducting external validation in multicenter, prospective cohorts to assess model generalizability and the stability of the decision threshold; performing implementation science research (e.g., stepped-wedge cluster randomized controlled trials) to evaluate the real-world impact of the online calculator on clinical decision-making and patient outcomes; designing basic experiments based on the hypotheses generated by the model (e.g., animal models, immune cell functional assays) to explore specific molecular mechanisms; and incorporating multimodal data to further improve predictive performance. All findings require independent validation before clinical translation can be considered.

## Data Availability

The raw data supporting the conclusions of this article will be made available by the authors, without undue reservation.

## Supplementary material

Supplementary Figure 3 Feature interaction analysis. Interaction plots examine how the effect of one clinical feature on PHN risk is modified by the level of another feature. Each panel shows the SHAP dependence of the x-axis feature, with smoothing lines (LOESS) colored according to tertiles (Low, Medium, High) of the interacting y-axis feature: blue lines represent low levels, yellow lines represent medium levels, and red lines represent high levels. Individual data points are colored by patient group (blue: control, red: PHN). Non-parallel lines across the three tertiles indicate significant interaction effects—that is, the influence of the x-axis feature on PHN risk depends on the level of the interacting feature. Parallel lines would suggest additive effects without interaction. The Spearman correlation coefficient between the x-axis feature and its SHAP value is provided in each subplot.
