# Supplementary figures and images for: Unraveling immune-inflammation-aging network interactions: an interpretable machine learning model predicts the risk of postherpetic neuralgia
Source: Front Immunol. 2026 Jun 12;17:1802320. doi: 10.3389/fimmu.2026.1802320 (PMC13303332; doi:10.3389/fimmu.2026.1802320)

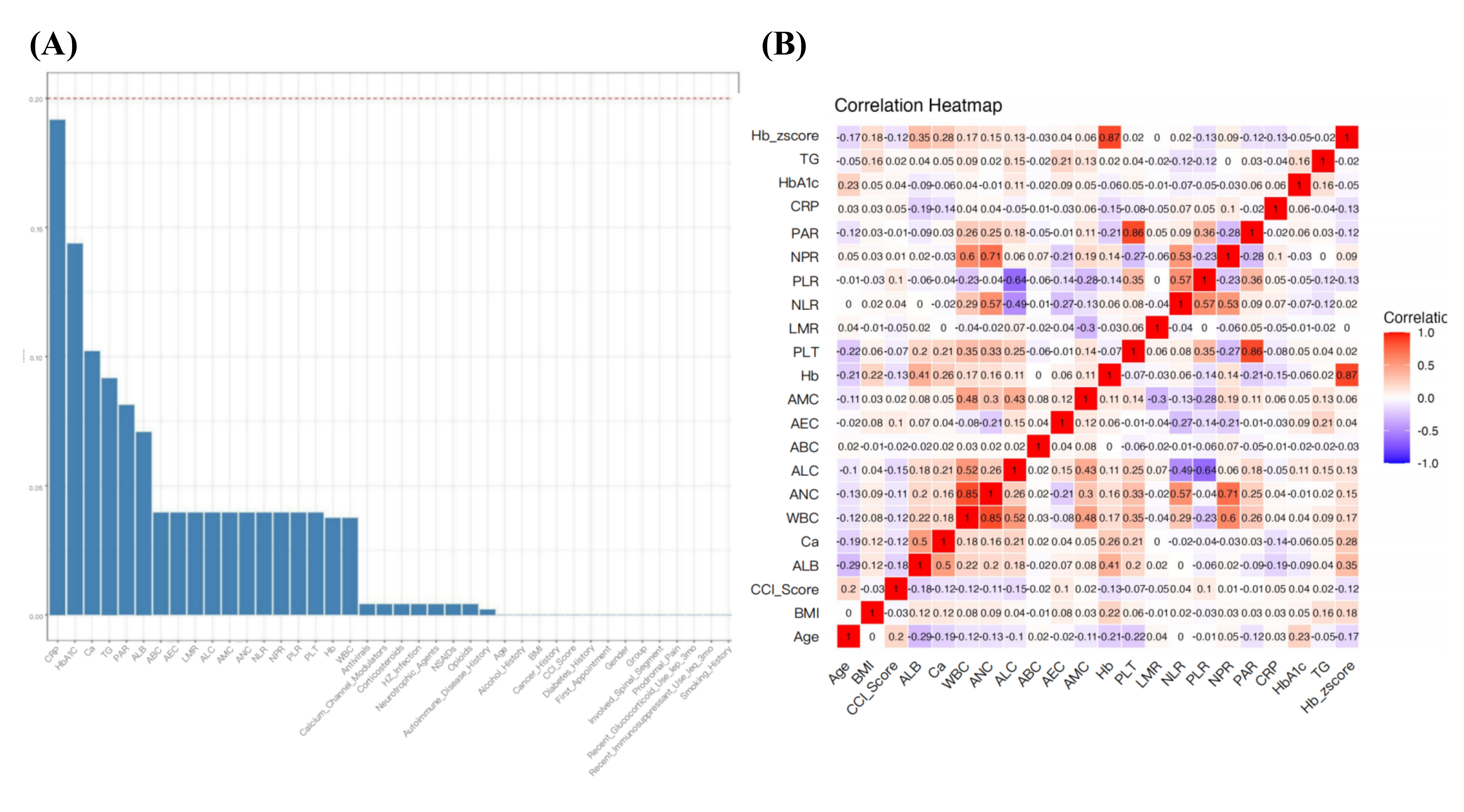

Supplement: Supplementary file 1 [file Image1.tif]

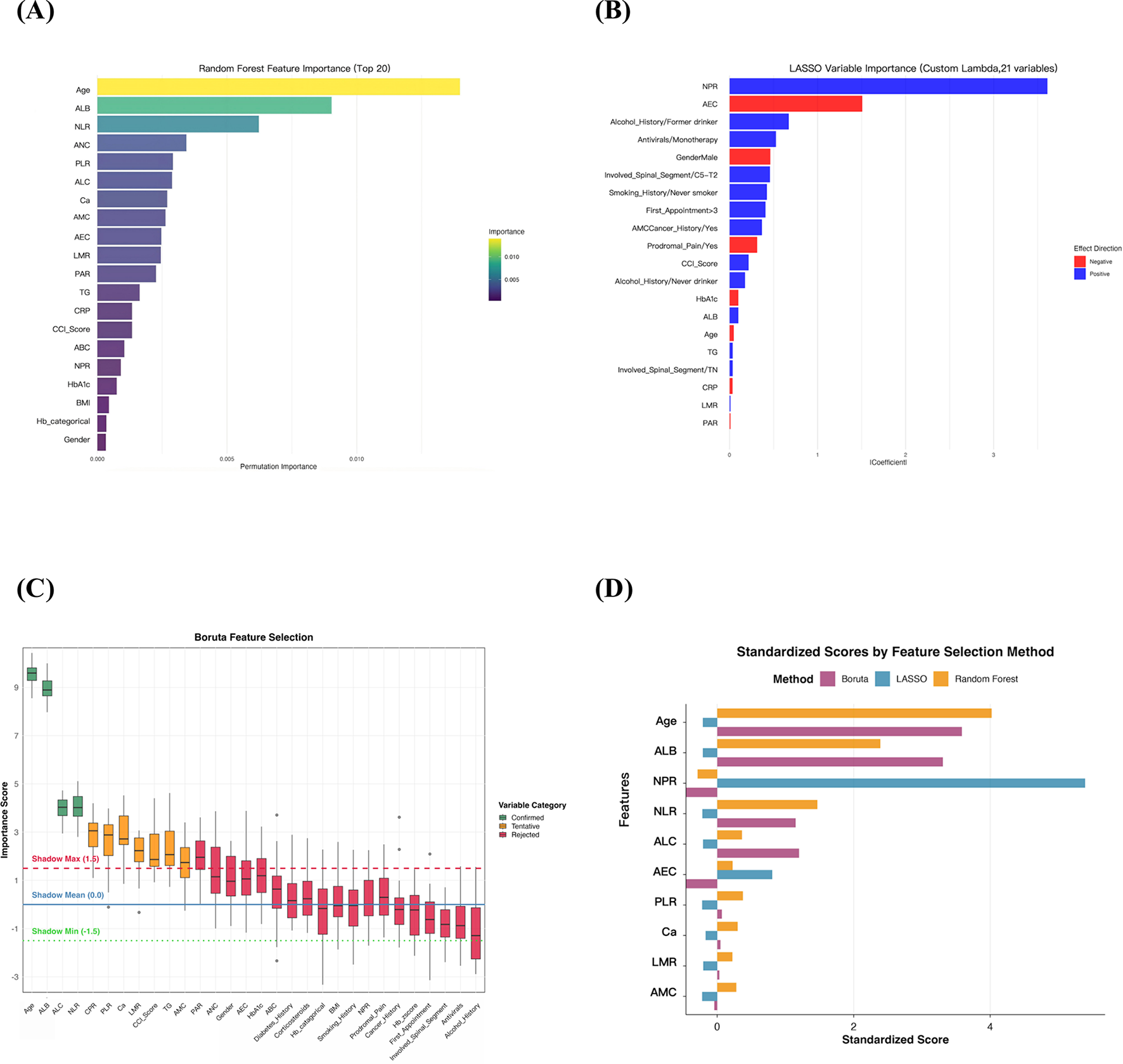

Supplement: Supplementary file 2 [file Image2.tif]

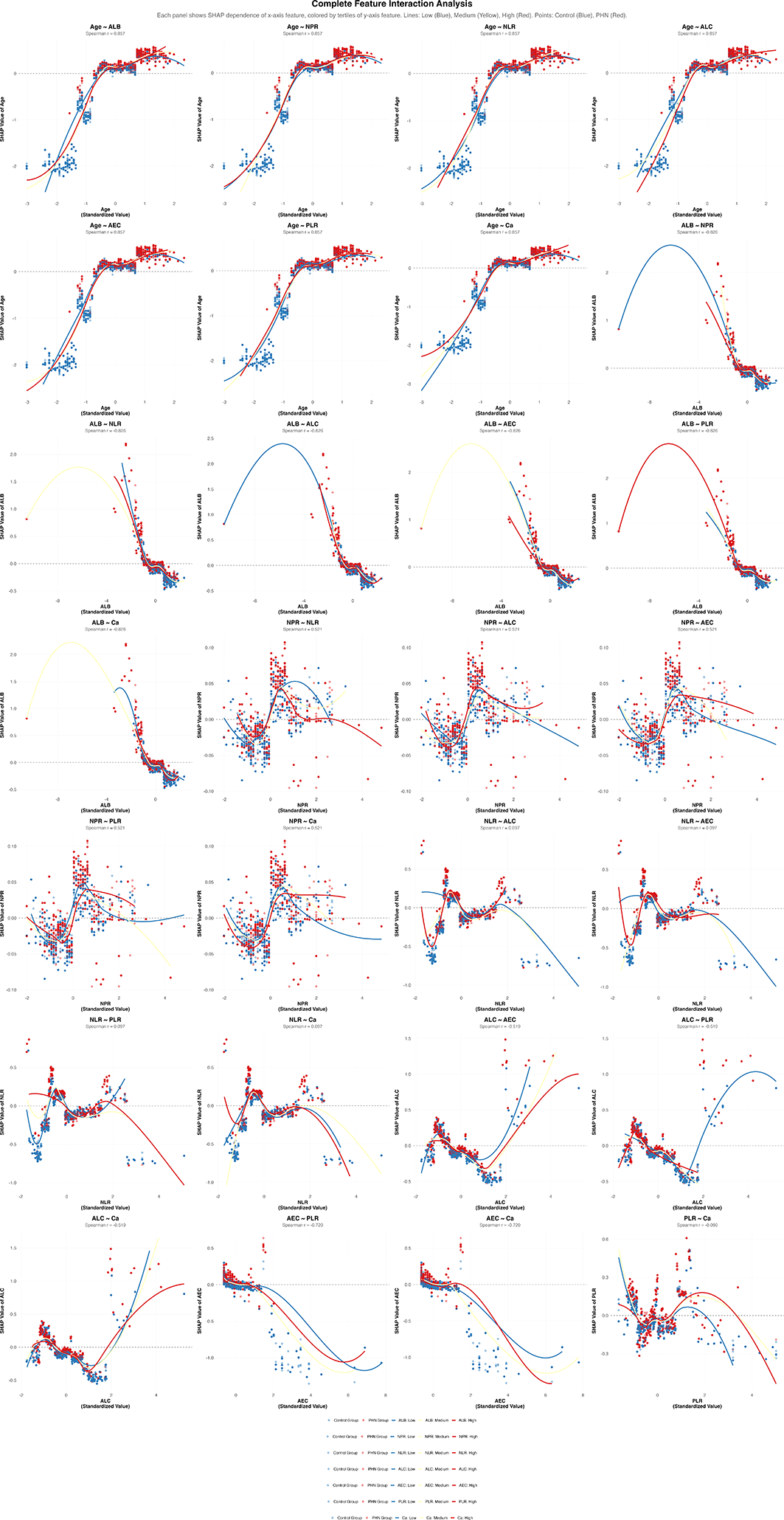

Supplement: Supplementary Figure 3 — Feature interaction analysis. Interaction plots examine how the effect of one clinical feature on PHN risk is modified by the level of another feature. Each panel shows the SHAP dependence of the x-axis feature, with smoothing lines (LOESS) colored according to tertiles (Low, Medium, High) of the interacting y-axis feature: blue lines represent low levels, yellow lines represent medium levels, and red lines represent high levels. Individual data points are colored by patient group (blue: control, red: PHN). Non-parallel lines across the three tertiles indicate significant interaction effects—that is, the influence of the x-axis feature on PHN risk depends on the level of the interacting feature. Parallel lines would suggest additive effects without interaction. The Spearman correlation coefficient between the x-axis feature and its SHAP value is provided in each subplot. [file Image3.tiff]
