# Supplementary material for: Unraveling immune-inflammation-aging network interactions: an interpretable machine learning model predicts the risk of postherpetic neuralgia
Source: Front Immunol. 2026 Jun 12;17:1802320. doi: 10.3389/fimmu.2026.1802320 (PMC13303332; doi:10.3389/fimmu.2026.1802320)
Supplement: Supplementary file 4 [file SupplementaryFile1.docx]

Supplementary Material

### S1. Multiple Imputation by Chained Equations (MICE) Specifications and Convergence Diagnostics

To handle missing data, we applied multiple imputation by chained equations (MICE) using the mice package in R (version 4.4.3). The imputation procedure was designed to preserve the multivariate distribution of the data and to account for the left-censored nature of C-reactive protein (CRP) values.

**Imputation parameters:** Ten imputations were generated (m = 10), with a maximum of 50 iterations per imputation (maxit = 50). Convergence was assessed by comparing means and standard deviations of the imputed data before and after each iteration. The random seed was fixed at 123 for reproducibility.

**Imputation methods by variable type:** For continuous non-censored variables, predictive mean matching (PMM) with a donor pool size of 5 was used. For binary variables, logistic regression (logreg) was applied. For left-censored CRP (detection limit = 3.11 mg/L), a two-stage Tobit-like imputation was implemented using a custom function mice.impute.tobit_custom. In the first stage, the censoring indicator crp_censored (1 = below detection limit, 0 = fully observed) was imputed using logistic regression. In the second stage, crp_value was imputed using a linear regression model based on fully observed cases, with predicted values constrained to ≤ 3.11 and non-negativity.

**Predictor matrix:** The predictor matrix was strictly defined to avoid logical inconsistencies. For example, crp_value was allowed to depend on crp_censored but was not allowed to predict itself; crp_censored was predicted by clinically relevant variables including group (PHN vs. control) and white blood cell count.

**Convergence diagnostics:** Convergence was verified by comparing the imputed data with the observed (non-imputed) values for all 19 continuous variables. The following criteria were assessed: mean and standard deviation differences (required to be <2% for all variables), median agreement, and Kolmogorov-Smirnov tests for distributional equivalence. The results of the convergence diagnostics are presented in Supplementary Table 2.

**Complete-case sensitivity analysis:** A complete-case sensitivity analysis was performed on the subset of patients with complete data for all eight core predictors (n = 415). The analysis compared model performance and feature importance between the complete-case dataset and the imputed dataset. The methods and results of this sensitivity analysis are provided in Supplementary Tables 11 and 12.

### S2. Integrated Feature Selection: Boruta, Random Forest, and LASSO

To identify the most predictive features for postherpetic neuralgia (PHN) while maintaining clinical interpretability, we employed an integrated feature selection strategy combining three complementary methods: the Boruta algorithm, random forest, and LASSO regression. All feature selection steps were performed exclusively within the training set (70% of the data) after the 7:3 random split of the complete imputed dataset (n = 480). The independent test set (30%) was not involved in any feature selection or hyperparameter tuning to prevent data leakage.

**Method-specific parameters:** For the random forest, we used 500 trees (ntree = 500), with the number of split variables at each node set to the square root of the total number of variables (mtry = √p). The minimum node size was set to 5, and importance was measured by MeanDecreaseAccuracy. For LASSO regression, we set α = 1 (L1 penalty) and determined the regularization strength λ via 10‑fold cross‑validation minimizing binomial deviance, selecting λ = 0.1039 using the one‑standard‑error rule for parsimony. Importance was defined as the absolute value of the standardized coefficient. For the Boruta algorithm, we used 100 maximum iterations (maxRuns = 100), a significance level α = 0.05, and the two‑sided Wilcoxon signed‑rank test against shadow features. Tentative features were discounted by a factor of 0.7, while rejected features were assigned an importance of 0.

**Normalization and composite scoring:** Because the raw importance scores from the three methods had different scales, we applied min‑max normalization to map each method’s scores to the [0,1] interval. We then computed a composite score using equal weights (1:1:1): Composite score = (Boruta_norm + LASSO_norm + RF_norm)/3.

**Optimal feature number determination:** Using the composite importance scores, we evaluated logistic regression performance with different numbers of top‑ranked features via 5‑fold cross‑validation on the training set. The number of features (k) was varied from 2 to 20 in steps of 2. The average accuracy peaked at k = 8 features, and beyond 8 features the accuracy plateaued or declined (*p* > 0.05 for improvement), suggesting a risk of overfitting. Therefore, the top 8 features were selected as the final predictor set.

**Stability analysis:** To assess the robustness of the final 8‑feature set, we performed two stability analyses. First, for random split stability, we fixed the first imputed dataset, used 100 different random seeds, each time re‑splitting the data into training/test sets (7:3) and running the complete feature selection pipeline independently. The selection frequency (number of times a feature appeared in the top 8) was recorded. Second, for bootstrap resampling stability, we drew 100 bootstrap samples (each sample size equal to the original training set) with replacement and applied the same feature selection procedure. Selection frequencies were recorded. The results of these stability analyses are presented in Supplementary Table 6.

**Weight sensitivity analysis:** We examined the impact of alternative weight assignments by varying the weight combinations: equal weights (1:1:1) as baseline; doubling one method [(2:1:1), (1:2:1), (1:1:2)]; and using a single method only [(1:0:0), (0:1:0), (0:0:1)]. The Jaccard similarity coefficient between the feature set selected under each weight combination and that selected under equal weights was calculated. The results of the weight sensitivity analysis are provided in Supplementary Table 7.
