# Supplementary material for: Unraveling immune-inflammation-aging network interactions: an interpretable machine learning model predicts the risk of postherpetic neuralgia
Source: Front Immunol. 2026 Jun 12;17:1802320. doi: 10.3389/fimmu.2026.1802320 (PMC13303332; doi:10.3389/fimmu.2026.1802320)
Supplement: Supplementary file 5 [file Table1.docx]

Supplementary Material

Table 1. **Definitions of Selected Predictive Variables**

| **Involved Nerve Segments** | As this is a retrospective study, some electronic medical records did not document the precise ganglia or nerves invaded by the herpes zoster virus. Therefore, based on symptomatic descriptions in the records, cases were classified into the following categories: TN, C1–4, C5–T2 (involving the shoulder girdle and upper limbs), T3–12, and LSR, which were assigned as 1, 2, 3, 4, and 5, respectively. |
| --- | --- |
| **Smoking History** | No smoking history was recorded as 0; former smoking (already quit) as 1; and current smoking as 2. |
| **Alcohol Consumption History** | No or occasional drinking was recorded as 0; former regular drinking (already quit) as 1; and current regular drinking as 2. |
| CCI-Score | Given the significant impact of diabetes, malignancies, and immune system disorders on both systemic immunity and neural involvement, and since previous studies have identified these as independent predictors of postherpetic neuralgia (PHN), they were excluded as separate predictive variables from the Charlson Comorbidity Index (CCI) score. The remaining comorbidities were scored according to the standard CCI scoring system. |
