# Supplementary material for: Unraveling immune-inflammation-aging network interactions: an interpretable machine learning model predicts the risk of postherpetic neuralgia
Source: Front Immunol. 2026 Jun 12;17:1802320. doi: 10.3389/fimmu.2026.1802320 (PMC13303332; doi:10.3389/fimmu.2026.1802320)
Supplement: Supplementary file 6 [file Table2.docx]

Supplementary Material

Table 2 Comparison of observed and imputed distributions for continuous variables

| Variable | Observed  Mean (SD) | Imputed  Mean (SD) | Observed Median | Imputed Median | KS *p*‑value |
| --- | --- | --- | --- | --- | --- |
| Age | 59.38 (14.40) | 59.38 (14.38) | 61 | 61 | > 0.99 |
| BMI | 24.97 (3.58) | 24.97 (3.58) | 24.71 | 24.71 | > 0.99 |
| CCI_Score | 0.18 (0.44) | 0.18 (0.43) | 0 | 0 | > 0.99 |
| HbA1c | 6.02 (0.94) | 6.06 (0.95) | 5.8 | 5.8 | 0.553 |
| ALB | 42.43 (4.09) | 42.46 (4.03) | 42.8 | 42.8 | > 0.99 |
| Ca | 2.30 (0.10) | 2.30 (0.10) | 2.3 | 2.3 | > 0.99 |
| TG | 1.15 (0.81) | 1.19 (0.88) | 0.91 | 0.91 | 0.993 |
| ANC | 4.73 (2.09) | 4.74 (2.10) | 4.44 | 4.45 | > 0.99 |
| ALC | 1.72 (0.74) | 1.71 (0.73) | 1.6 | 1.6 | > 0.99 |
| ABC | 0.04 (0.23) | 0.04 (0.23) | 0.02 | 0.02 | > 0.99 |
| AEC | 0.06 (0.09) | 0.06 (0.09) | 0.02 | 0.02 | > 0.99 |
| AMC | 0.51 (0.22) | 0.51 (0.22) | 0.5 | 0.5 | > 0.99 |
| Hb | 135.58 (15.49) | 135.64 (15.50) | 136 | 136 | > 0.99 |
| LMR | 4.09 (5.48) | 4.07 (5.38) | 3.46 | 3.42 | > 0.99 |
| NLR | 3.12 (1.69) | 3.12 (1.68) | 2.72 | 2.73 | > 0.99 |
| PLR | 145.87 (69.04) | 145.66 (69.64) | 132.47 | 132.28 | > 0.99 |
| NPR | 0.023 (0.010) | 0.023 (0.010) | 0.021 | 0.021 | > 0.99 |
| PAR | 5.12 (1.37) | 5.10 (1.36) | 5.04 | 5.04 | > 0.99 |
| crp_value | 5.20 (6.90) | 5.05 (6.35) | 3.11 | 3.11 | > 0.99 |
