# Supplementary material for: Unraveling immune-inflammation-aging network interactions: an interpretable machine learning model predicts the risk of postherpetic neuralgia
Source: Front Immunol. 2026 Jun 12;17:1802320. doi: 10.3389/fimmu.2026.1802320 (PMC13303332; doi:10.3389/fimmu.2026.1802320)
Supplement: Supplementary file 7 [file Table3.docx]

Supplementary Material

Table 2. **Deleted Variables**

| **Zero- or Near-Zero-Variance Variable** | Highly Correlated Variable Pairs | Ultimately Deleted Variables |
| --- | --- | --- |
| Use of glucocorticoids within ≤3 months | PAR\PLT | Use of glucocorticoids within ≤3 months |
| Use of immunosuppressants within ≤3 months | Hb\Hb_zscore | Use of immunosuppressants within ≤3 months |
| History of autoimmune disease | WBC\ Neutrophil_Count | History of autoimmune disease |
| Occurrence of herpes infection |  | Occurrence of herpes infection |
| Calcium channel modulators |  | Calcium channel modulators |
| Neurotrophic drugs |  | Neurotrophic drugs |
| NSAIDs |  | NSAIDs |
| Opioids |  | Opioids |
|  |  | WBC\PLT\Hb |
