# Supplementary material for: Unraveling immune-inflammation-aging network interactions: an interpretable machine learning model predicts the risk of postherpetic neuralgia
Source: Front Immunol. 2026 Jun 12;17:1802320. doi: 10.3389/fimmu.2026.1802320 (PMC13303332; doi:10.3389/fimmu.2026.1802320)
Supplement: Supplementary file 8 [file Table4.docx]

Supplementary Material

Table 4. Hyperparameter Settings for Feature Selection Methods

| Method | Parameter | Setting Value | Description / Rationale |
| --- | --- | --- | --- |
| Random Forest | Number of trees (ntree) | 500 | Ensemble learning based on 500 decision trees. |
|  | Number of split variables (mtry) | √p (where p is the total number of variables) | The square root of the total variable count is randomly considered for each split. |
|  | Minimum node size (nodesize) | 5 | The minimum number of samples required in a terminal node to control tree growth. |
| LASSO Regression | Type of regularization (α) | 1 (LASSO) | α=1 corresponds to LASSO regression, which performs L1 penalty for feature selection. |
|  | Regularization strength (λ) | 0.1039 | Determined via 10-fold cross-validation minimizing the binomial deviance. |
|  | Criterion for λ selection | 1 standard error rule | Selects the most parsimonious model within one standard error of the minimum deviance, balancing complexity and performance. |
| Boruta Algorithm | Maximum iterations (maxRuns) | 100 | Ensures sufficient convergence of the algorithm. |
|  | Significance level (α) | 0.05 | The threshold for the statistical test of feature importance. |
|  | Decision criterion | Two-sided Wilcoxon signed-rank test (against shadow features) | Compares the importance of real features with that of randomly permuted shadow features. |
| Integrated Evaluation | Standardized Method for Weight Allocation | Equal Weight (1:1:1)  Min-Max Normalization | Equal weights for the three methods.  Map to (0, 1) |
| Cross-Validation | Folds & evaluation strategy | 5-fold cross-validation | Model performance with different k values (number of selected features) was evaluated via 5-fold CV on the training set to determine the optimal k. |
|  | Feature number k | 2 to 20 (step size: 2) | The range of candidate numbers of features to be selected. |
|  | Optimal k | 8 | The final number of features selected, offering a balance between model per |
