# Supplementary material for: Unraveling immune-inflammation-aging network interactions: an interpretable machine learning model predicts the risk of postherpetic neuralgia
Source: Front Immunol. 2026 Jun 12;17:1802320. doi: 10.3389/fimmu.2026.1802320 (PMC13303332; doi:10.3389/fimmu.2026.1802320)
Supplement: Supplementary file 9 [file Table5.docx]

Supplementary Material

Table **5**.**  Hyperparameter Configurations for the Six Predictive Models**

| Model | Hyperparameter | Search Space in Nested CV (Coarse Grid) | Final Model Parameters (Main Text Table 4) | Remark |
| --- | --- | --- | --- | --- |
| XGBoost | nrounds | {50, 100} | 80 | Final value within grid range |
|  | max_depth | {2, 3} | 2 | Within grid range |
|  | eta | {0.01, 0.05} | 0.03 | Fine-tuned between the two points |
|  | gamma | {1, 3} | 2 | Fine-tuned between the two points |
|  | colsample_bytree | {0.6, 0.7} | 0.6* | Selected from the grid |
|  | min_child_weight | {3, 5} | 4* | Fine-tuned between the two points |
|  | subsample | {0.6, 0.7} | 0.65* | Fine-tuned between the two points |
| LightGBM | learning_rate | {0.01 (fixed)} | 0.01 | Fixed value |
|  | num_leaves | {7 (fixed)} | 7 | Fixed value |
|  | max_depth | {3 (fixed)} | 3 | Fixed value |
|  | lambda_l1 | {0.5, 1.0} | 0.5 | Grid search |
|  | lambda_l2 | {1.0 (fixed)} | Fixed but not listed |  |
|  | feature_fraction | {0.6 (fixed)} | Fixed but not listed |  |
| NN | size | {3, 5, 7} | 5 | Grid search |
|  | decay | {0.001, 0.01, 0.1} | 0.01 | Grid search |
| LR | alpha | {0, 0.5, 1} | 0.5 | Grid search |
|  | lambda | {0.001, 0.01, 0.1} | 0.01 | Grid search |
| RF | mtry | {1, 2} | 2 | Grid search |
|  | ntree | {300 (fixed)} | 300 | Fixed |
|  | nodesize | {10 (fixed)} | 10 | Fixed |
|  | maxnodes | {15 (fixed)} | 15 | Fixed |
| SVM | C (regularization) | 2^seq(-5, 0, length=5) | 0.125 | Grid search |
|  | sigma (RBF kernel) | 2^seq(-9, -3, length=5) | 0.03125 | Grid search |
