# Supplementary material for: Unraveling immune-inflammation-aging network interactions: an interpretable machine learning model predicts the risk of postherpetic neuralgia
Source: Front Immunol. 2026 Jun 12;17:1802320. doi: 10.3389/fimmu.2026.1802320 (PMC13303332; doi:10.3389/fimmu.2026.1802320)
Supplement: Supplementary file 10 [file Table6.docx]

Supplementary Material

Table 6. Comparison of Feature Selection Frequencies in the Top 8 under Two Perturbation Resampling Methods

| Feature | Bootstrap Frequency（100） | Random  Frequency（100） | Mean Frequency | Overall Stability |
| --- | --- | --- | --- | --- |
| Age | 1 | 1 | 1 | Extremely High |
| ALB | 0.95 | 0.91 | 0.93 | Extremely High |
| ALC | 0.68 | 0.81 | 0.745 | High |
| AEC | 0.44 | 0.75 | 0.595 | Moderate |
| NLR | 0.35 | 0.63 | 0.49 | Relatively Low |
| Ca | 0.66 | 0.23 | 0.445 | Relatively Low |
| NPR | 0.49 | 0.23 | 0.36 | Relatively Low |
| PLR | 0.28 | 0.32 | 0.3 | Relatively Low |

*Note: The classification of overall stability is based on the mean frequency:Mean_Frequency >= 0.9 ~ “Extremely High”, Mean_Frequency >= 0.7 ~ “High”, Mean_Frequency >= 0.5 ~ "Moderate”, Mean_Frequency >= 0.3 ~ "Relatively Low".*
