# Supplementary material for: Unraveling immune-inflammation-aging network interactions: an interpretable machine learning model predicts the risk of postherpetic neuralgia
Source: Front Immunol. 2026 Jun 12;17:1802320. doi: 10.3389/fimmu.2026.1802320 (PMC13303332; doi:10.3389/fimmu.2026.1802320)
Supplement: Supplementary file 11 [file Table7.docx]

Supplementary Material

Table 7. Jaccard Similarity between Different Weight Combinations and Equal Weights

| Weight Combination (Boruta : LASSO : RF) | Jaccard Similarity |
| --- | --- |
| 1:1:1 (Baseline) | 1.000 |
| 2:1:1 (Boruta doubled) | 0.778 |
| 1:2:1 (LASSO doubled) | 1.000 |
| 1:1:2 (RF doubled) | 1.000 |
| 1:0:0 (Boruta only) | 0.600 |
| 0:1:0 (LASSO only) | 0.143 |
| 0:0:1 (RF only) | 0.778 |
