# Supplementary material for: Unraveling immune-inflammation-aging network interactions: an interpretable machine learning model predicts the risk of postherpetic neuralgia
Source: Front Immunol. 2026 Jun 12;17:1802320. doi: 10.3389/fimmu.2026.1802320 (PMC13303332; doi:10.3389/fimmu.2026.1802320)
Supplement: Supplementary file 12 [file Table8.docx]

Supplementary Material

Table 8. Specific Net Benefit Values at Key Thresholds

| Threshold Probability | Net Benefit (XGBoost Calibrated) | Net Benefit (Treat All) | Net Benefit (Treat None) |
| --- | --- | --- | --- |
| 0.00 (0%) | 0.233 | 0.233 | 0 |
| 0.01 (1%) | 0.227 | 0.225 | 0 |
| 0.05 (5%) | 0.21 | 0.193 | 0 |
| 0.10 (10%) | 0.194 | 0.148 | 0 |
| 0.15 (15%) | 0.177 | 0.098 | 0 |
| 0.20 (20%) | 0.159 | 0.041 | 0 |
| 0.25 (25%) | 0.15 | 0 | 0 |
| 0.275 (27.5%) | 0.146* | 0 | 0 |
| 0.30 (30%) | 0.139 | 0 | 0 |
| 0.35 (35%) | 0.129 | 0 | 0 |
| 0.40 (40%) | 0.121 | 0 | 0 |
| 0.45 (45%) | 0.109 | 0 | 0 |
| 0.50 (50%) | 0.103 | 0 | 0 |
