# Supplementary material for: Unraveling immune-inflammation-aging network interactions: an interpretable machine learning model predicts the risk of postherpetic neuralgia
Source: Front Immunol. 2026 Jun 12;17:1802320. doi: 10.3389/fimmu.2026.1802320 (PMC13303332; doi:10.3389/fimmu.2026.1802320)
Supplement: Supplementary file 13 [file Table9.docx]

Supplementary Material

Table 9. Specific Net Benefit Values at Key Thresholds

| Feature_X | Feature_Y | Interaction_p | Interaction_F | Max_SHAP_Diff | Low_Group_Mean | Medium_Group_Mean | High_Group_Mean |
| --- | --- | --- | --- | --- | --- | --- | --- |
| Age | ALB | 7.43×10⁻94 | 224.343 | 0.437 | 0.054 | -0.124 | -0.382 |
|  |  |  |  |  |  |  |  |
| Age | NPR | 0.102 | 2.28 | 0.008 | -0.15 | -0.143 | -0.152 |
| Age | NLR | 8.10×10⁻^17^ | 37.34 | 0.049 | -0.158 | -0.169 | -0.12 |
|  |  |  |  |  |  |  |  |
| Age | ALC | 1.97×10⁻83 | 198.22 | 0.268 | -0.027 | -0.127 | -0.295 |
|  |  |  |  |  |  |  |  |
| Age | AEC | 0.062 | 2.784 | 0.113 | -0.099 | -0.212 | -0.168 |
| Age | PLR | 2.29×10⁻4 | 8.396 | 0.09 | -0.1 | -0.19 | -0.154 |
|  |  |  |  |  |  |  |  |
| Age | Ca | 2.27×10⁻18 | 43.317 | 0.176 | -0.078 | -0.124 | -0.254 |
|  |  |  |  |  |  |  |  |
| ALB | NPR | 4.61×10⁻18 | 40.254 | 0.057 | 0.018 | -0.039 | -0.03 |
|  |  |  |  |  |  |  |  |
| ALB | NLR | 2.30×10⁻147 | 362.639 | 0.048 | -0.015 | -0.043 | 0.006 |
|  |  |  |  |  |  |  |  |
| ALB | ALC | 2.84×10⁻6 | 12.805 | 0.138 | 0.071 | -0.054 | -0.067 |
|  |  |  |  |  |  |  |  |
| ALB | AEC | 6.28×10⁻64 | 151.78 | 0.086 | 0.021 | 0 | -0.065 |
|  |  |  |  |  |  |  |  |
| ALB | PLR | 2.75×10⁻12 | 26.766 | 0.058 | -0.036 | -0.035 | 0.022 |
| ALB | Ca | 3.37×10⁻162 | 402.317 | 0.277 | 0.122 | -0.04 | -0.155 |
| NPR | NLR | 5.70×10⁻19 | 184.64 | 0.019 | -0.012 | -0.002 | 0.007 |
| NPR | ALC | 4.71×10⁻19 | 42.573 | 0.005 | -0.003 | -0.005 | 0.001 |
| NPR | AEC | 2.88×10⁻4 | 8.171 | 0.018 | 0.005 | 0.004 | -0.013 |
| NPR | PLR | 2.38×10⁻19 | 43.27 | 0.014 | 0.007 | -0.007 | -0.007 |
| NPR | Ca | 5.72×10⁻48 | 111.297 | 0.004 | -0.004 | 0 | -0.003 |
| NLR | ALC | 1.31×10⁻168 | 419.557 | 0.083 | -0.022 | -0.009 | -0.091 |
| NLR | AEC | 1.21×10⁻86 | 209.501 | 0.093 | 0.023 | -0.021 | -0.071 |
| NLR | PLR | 4.88×10⁻122 | 296.312 | 0.085 | -0.086 | -0.001 | -0.031 |
| NLR | Ca | 0.048 | 3.048 | 0.038 | -0.021 | -0.059 | -0.042 |
| ALC | AEC | 1.05×10⁻11 | 25.462 | 0.008 | -0.041 | -0.049 | -0.042 |
| ALC | PLR | 2.66×10⁻223 | 571.608 | 0.129 | -0.068 | -0.079 | 0.051 |
| ALC | Ca | 2.15×10⁻26 | 59.841 | 0.008 | -0.03 | -0.035 | -0.026 |
| AEC | PLR | 2.13×10⁻16 | 36.361 | 0.045 | -0.033 | -0.022 | 0.012 |
| AEC | Ca | 5.40×10⁻63 | 147.769 | 0.04 | 0.009 | -0.025 | -0.031 |
| PLR | Ca | 0.011 | 4.506 | 0.012 | -0.018 | -0.007 | -0.012 |
