# Supplementary material for: Unraveling immune-inflammation-aging network interactions: an interpretable machine learning model predicts the risk of postherpetic neuralgia
Source: Front Immunol. 2026 Jun 12;17:1802320. doi: 10.3389/fimmu.2026.1802320 (PMC13303332; doi:10.3389/fimmu.2026.1802320)
Supplement: Supplementary file 14 [file Table10.docx]

Supplementary Material

Table 10. Stratified correlation patterns and interaction strength classification for predictor pairs

| Feature_X | Feature_Y | r_low | r_medium | r_high | r_range | Interaction_Type |
| --- | --- | --- | --- | --- | --- | --- |
| NLR | ALC | -0.258873345 | -0.0448565 | 0.591763216269836 | 0.85063656082428 | Strong Synergistic |
| NLR | AEC | -0.297875392 | 0.302858507915633 | 0.490047075349547 | 0.787922467175579 | Strong Synergistic |
| NLR | PLR | 0.547881478583216 | -0.040300239 | -0.199985749 | 0.747867227116627 | Strong Synergistic |
| ALC | PLR | -0.174920237 | -0.762661355 | -0.278977051 | 0.587741117663006 | Strong Synergistic |
| ALC | Ca | -0.561265151 | -0.666678714 | -0.3106889 | 0.355989814529989 | Strong Synergistic |
| NPR | Ca | 0.346491631135272 | 0.594473278168795 | 0.65566555769253 | 0.309173926557258 | Strong Synergistic |
| NPR | NLR | 0.452701892921142 | 0.547137122360949 | 0.251327714165607 | 0.295809408195342 | Moderate Synergistic |
| NPR | ALC | 0.343125999730035 | 0.630183912170808 | 0.599231232019758 | 0.287057912440773 | Moderate Synergistic |
| AEC | PLR | -0.767376612 | -0.753940839 | -0.48095926 | 0.286417351140614 | Moderate Synergistic |
| NLR | Ca | -0.008692286 | 0.196172231395643 | 0.0864452358067515 | 0.204864517056069 | Moderate Synergistic |
| NPR | PLR | 0.457500615914136 | 0.556630438893083 | 0.406178501570037 | 0.150451937323046 | Moderate Synergistic |
| NPR | AEC | 0.422952364115588 | 0.486508446316755 | 0.558567036490145 | 0.135614672374557 | Weak Synergistic |
| AEC | Ca | -0.677748623 | -0.686364427 | -0.808070746 | 0.130322123370402 | Weak Synergistic |
| Age | ALB | 0.781208562780157 | 0.83543878223265 | 0.899127466893076 | 0.117918904112919 | Weak Synergistic |
| Age | PLR | 0.783125359458359 | 0.871437257259389 | 0.885043787235078 | 0.101918427776719 | Weak Synergistic |
| ALB | Ca | -0.860809554 | -0.76412085 | -0.807915293 | 0.0966887034804641 | Weak Synergistic |
| PLR | Ca | -0.128495047 | -0.045229361 | -0.09581691 | 0.083265685302323 | Weak Synergistic |
| ALC | AEC | -0.528893456 | -0.488837243 | -0.452449012 | 0.076444444101093 | Weak Synergistic |
| ALB | PLR | -0.874650317 | -0.829243012 | -0.801975122 | 0.072675194637822 | Weak Synergistic |
| Age | NPR | 0.818884955488889 | 0.889560735415006 | 0.844169020367949 | 0.0706757799261171 | Weak Synergistic |
| Age | Ca | 0.835746277505758 | 0.819170404821295 | 0.873824623897008 | 0.054654219075713 | Weak Synergistic |
| ALB | NLR | -0.853117395 | -0.799875114 | -0.83131756 | 0.0532422810843081 | Weak Synergistic |
| ALB | ALC | -0.813065052 | -0.820348942 | -0.86383685 | 0.050771798270503 | Weak Synergistic |
| ALB | NPR | -0.801683298 | -0.836310401 | -0.844282921 | 0.042599622910899 | Additive |
| Age | NLR | 0.83248406515611 | 0.870625199169883 | 0.854816295800942 | 0.0381411340137731 | Additive |
| Age | ALC | 0.834257197951121 | 0.86892700098764 | 0.854124482341558 | 0.034669803036519 | Additive |
| Age | AEC | 0.854047313857567 | 0.866772554361173 | 0.837209895642245 | 0.0295626587189279 | Additive |
| ALB | AEC | -0.840347352 | -0.839953302 | -0.817299889 | 0.023047462642803 | Additive |
