# Supplementary material for: Unraveling immune-inflammation-aging network interactions: an interpretable machine learning model predicts the risk of postherpetic neuralgia
Source: Front Immunol. 2026 Jun 12;17:1802320. doi: 10.3389/fimmu.2026.1802320 (PMC13303332; doi:10.3389/fimmu.2026.1802320)
Supplement: Supplementary file 15 [file Table11.docx]

| Group | Sample Size | Mean Number of Missing Values (SD) | Proportion of Excluded Samples |
| --- | --- | --- | --- |
| Control | 368 | 0.435 (1.39) | 58/65 (89.2%) |
| PHN | 112 | 0.161 (0.886) | 7/65 (10.8%) |
| Between-group comparison |  | p = 0.0093 (Wilcoxon) | — |

Supplementary Material

Table 10.  Analysis of Missing Mechanism
