# Supplementary material for: Unraveling immune-inflammation-aging network interactions: an interpretable machine learning model predicts the risk of postherpetic neuralgia
Source: Front Immunol. 2026 Jun 12;17:1802320. doi: 10.3389/fimmu.2026.1802320 (PMC13303332; doi:10.3389/fimmu.2026.1802320)
Supplement: Supplementary file 16 [file Table12.docx]

Supplementary Material

Table 12. Performance Comparison of XGBoost between Complete Case Analysis (CCA) and Multiple Imputation Primary Analysis

| Metric | CCA (n=415) | Primary Analysis (n=480, after imputation) |
| --- | --- | --- |
| AUC | 0.657 ± 0.051 | 0.919 ± 0.009 |
| Accuracy | 0.636 ± 0.070 | 0.874 |
| Sensitivity | 0.562 ± 0.103 | 0.836 ± 0.037 |
| Specificity | 0.661 ± 0.081 | 0.831 ± 0.020 |
