# Supplementary material for: Unraveling immune-inflammation-aging network interactions: an interpretable machine learning model predicts the risk of postherpetic neuralgia
Source: Front Immunol. 2026 Jun 12;17:1802320. doi: 10.3389/fimmu.2026.1802320 (PMC13303332; doi:10.3389/fimmu.2026.1802320)
Supplement: Supplementary file 17 [file Table13.docx]

Supplementary Material

**Table** 13. **Coefficient of Variation of Feature Importance**

| variable | mean importance | sd importance | cv importance | n_folds |
| --- | --- | --- | --- | --- |
| Age | 0.337 | 0.007 | 0.021 | 5 |
| NLR | 0.150 | 0.004 | 0.024 | 5 |
| ALC | 0.142 | 0.0033 | 0.023 | 5 |
| ALB | 0.130 | 0.005 | 0.037 | 5 |
| PLR | 0.084 | 0.002 | 0.029 | 5 |
| AEC | 0.077 | 0.001 | 0.019 | 5 |
| Ca | 0.057 | 0.002 | 0.042 | 5 |
| NPR | 0.023 | 0.007 | 0.029 | 5 |
