# Supplementary material for: Unraveling immune-inflammation-aging network interactions: an interpretable machine learning model predicts the risk of postherpetic neuralgia
Source: Front Immunol. 2026 Jun 12;17:1802320. doi: 10.3389/fimmu.2026.1802320 (PMC13303332; doi:10.3389/fimmu.2026.1802320)
Supplement: Supplementary file 18 [file Table14.docx]

Supplementary Material

**Table** 14A. **Comparison with Previous Studies on PHN Prediction Models**

| **Study** | **Year** | **Sample Size** | **Feature Selection** | **Model** | **Validation Strategy** |
| --- | --- | --- | --- | --- | --- |
| **[1](1)** | **2026** | **846** | **LASSO + Boruta** | **XGBoost** | **External validation** |
| **[2](2)** | **2026** | **8,878** | **NR** | **XGBoost** | **Train/Test split** |
| **[3](3)** | **2024** | **209** | **Univariate+Multivariate** | **Logistic (Nomogram)** | **Bootstrap** |
| **[4](4)** | **2024** | **174** | **Univariate+Multivariate** | **Logistic (Nomogram)** | **NR** |
| **[5](5)** | **2026** | **722** | **Stepwise** | **Logistic** | **Train/Test (7:3)** |
| **[6](6)** | **2026** | **211** | **Univariate** | **ROC (no ML)** | **NR** |
| **[7](7)** | **2022** | **60** | **LASSO** | **SVM** | **Cross-validation** |
| **[8](8)** | **2025** | **260** | **Univariate + Multivariate** | **Logistic (Nomogram)** | **Train/Test (7:3)** |
| This study | **2025** | **480** | **Boruta+RF+LASSO (integration)** | **XGBoost** | **Nested cross validation** |

**Table** 14B. **Comparison with Previous Studies on PHN Prediction Models**

| Study | Year | Key Predictors | AUC | Key Limitation |
| --- | --- | --- | --- | --- |
| **[1](1)** | **2026** | **Age, diabetes, prodromal pain, acute pain severity, antiviral timing** | **0.84** | **Subjective (NRS)** |
| **[2](2)** | **2026** | **Age, female sex, prior HZ, cancer, immunosuppressants, antidepressants, initial pain, NLR** | **0.787** | **Severe PHN only** |
| **[3](3)** | **2024** | **Age, NRS-11, PLR** | **0.787** | **Small sample, subjective (NRS)** |
| **[4](4)** | **2024** | **Age>50, female, prodromal pain, rash area, VAS score** | **0.81** | **Subjective (VAS)** |
| **[5](5)** | **2026** | **Age, duration, prodromal pain, VZV-IgM, RDW-CV, urea, Na** | **0.733** | **Moderate AUC** |
| **[6](6)** | **2026** | **Clinical + sMRI (12 brain regions)** | **0.748** | **Expensive imaging, no ML** |
| **[7](7)** | **2022** | **Metabolomics + proteomics** | **0.87-0.907** | **Very small sample (n=60), expensive** |
| **[8](8)** | **2025** | **Age, CD4+/CD8+, Treg, IL-6, TNF-α, IL-10** | **0.802** | **High cost (flow cytometry, ELISA)** |
| **This study** | **2025** | **Age, NLR, ALC, ALB, PLR, AEC, Ca, NPR** | **0.919** | **Need external validation** |

# **Reference**

**1. Chen X, Lin X, Lin C, Lin C, Lin J, Li J. Development and validation of a machine learning model for predicting postherpetic neuralgia risk. Front Neurol. 2026;17:1775957: 1775957. eng.Epub 2026/04/16. doi:10.3389/fneur.2026.1775957. Cited in: PubMed; PMID 42079829.**

**2. Park SJ, Han J, Choi JB, Min SK, Park J, Choi S. Deciphering risk factors for severe postherpetic neuralgia in patients with herpes zoster: an interpretable machine learning approach. Reg Anesth Pain Med. 2026;51(4):429-36: 429-36. eng.Epub 2026/04/02. doi:10.1136/rapm-2024-106003. Cited in: PubMed; PMID 39779279.**

**3. Cai M, Yin J, Zeng Y, Liu H, Jin Y. A Prognostic Model Incorporating Relevant Peripheral Blood Inflammation Indicator to Predict Postherpetic Neuralgia in Patients with Acute Herpes Zoster. J Pain Res. 2024;17:2299-309: 2299-309. eng.Epub 2024/07/01. doi:10.2147/jpr.S466939. Cited in: PubMed; PMID 38974827.**

**4. Hu HM, Mao P, Liu X, Zhang YJ, Li C, Zhang Y, et al. A Nomogram Model for Predicting Postherpetic Neuralgia in Patients with Herpes Zoster: A Prospective Study. Pain Physician. 2024;27(8):E843-e50: E843-e50. eng.Epub. Cited in: PubMed; PMID 39621984.**

**5. Wang J, Yao Y, Wang H, Chen J, Huang S, Li S, et al. A Multicenter Prospective Study to Develop a Prediction Model for Postherpetic Neuralgia Using Clinical and Laboratory Indicators. Pain Ther. 2026. eng.Epub 2026/04/22. doi:10.1007/s40122-026-00836-9. Cited in: PubMed; PMID 42020636.**

**6. Wu D, Peng B, Hua Y, Geng W, Huang B, Lu S, et al. Prediction of Postherpetic Neuralgia in Patients with Acute and Subacute Herpetic Neuralgia Using Structural Magnetic Resonance Imaging: A Retrospective Study. Pain Ther. 2026;15(1):269-89: 269-89. eng.Epub 2025/12/07. doi:10.1007/s40122-025-00796-6. Cited in: PubMed; PMID 41353704.**

**7. Zhou R, Li J, Zhang Y, Xiao H, Zuo Y, Ye L. Characterization of plasma metabolites and proteins in patients with herpetic neuralgia and development of machine learning predictive models based on metabolomic profiling. Front Mol Neurosci. 2022;15:1009677: 1009677. eng.Epub 2022/10/06. doi:10.3389/fnmol.2022.1009677. Cited in: PubMed; PMID 36277496.**

**8. Liu L, Chen S. Construction of influencing factors and nomogram prediction model for post-herpetic neuralgia based on T cell function and inflammatory factors. Front Med (Lausanne). 2025;12:1619157: 1619157. eng.Epub 2025/06/18. doi:10.3389/fmed.2025.1619157. Cited in: PubMed; PMID 40606470.**
